# Supplementary material for: Metabolic versatility and nitrate reduction pathways of a new thermophilic bacterium of the Deferrivibrionaceae: Deferrivibrio metallireducens sp. nov isolated from hot sediments of Vulcano Island, Italy
Source: PLoS One. 2025 Mar 11;20(3):e0315093. doi: 10.1371/journal.pone.0315093 (PMC11896075; doi:10.1371/journal.pone.0315093)
Supplement: Figure S1 — The evolutionary history was inferred using the Maximum Likelihood method and General Time Reversible model. The percentage of trees in which the associated taxa clustered is shown next to the branches. This analysis involved 23 nucleotide sequences. The outgroup was composed of the following type species: Thermanaeromonas toyohensis ToBE (AB062280), Moorella humiferrea 64 FGQ (GQ872425), Desulfovibrio indonesiensis Ind 1 (Y09504), Desulfovibrio marinus E-2 (DQ365924), Desulfonatronum cooperativum Z-7999 (AY725424), Desulfonatronum thioautotrophicum ASO4-1 (FJ469577), Desulfonatronum thiodismutans MLF1 (AF373920), Desulfonatronum lacustre DSM 10312 (AF418171), Desulfonatronum buryatense Ki5 (KC417374). There were a total of 2753 positions in the final dataset. Evolutionary analyses were conducted in MEGA 7 (35). Taxonomy is based on LPSN. Figure S2. Polar lipids of strain V6Fe1T. The polar lipid profile of strain V6Fe1T consisted of glycolipids, phospholipids, phosphatidylglycerol, phosphatidylethanolamine, and diphosphatidylglycerol. Figure S3. Venn diagram of protein orthologs shared by the assembly of D. autotrophicus chromosome and plasmid of D. desulfuricans and C. nitroreducens. Orthology analysis was performed with OrthoVenn2 web server using 0.01 blast e-value and 1.5 orthoMCL grain value. Numbers indicate shared or unique protein clusters and singleton proteins. Table S1. Primers used in this study, designed using Primer3 software version 4.1.0, targeting cymA, hcp, napA, norV, nrfD (present in two copies), nrfA genes encoding respectively a cytoplasmic membrane electron transport protein involved in nitrate reduction, a hydroxylamine reductase, a periplasmic nitrate reductase, a nitric oxide reductase, an integral transmembrane protein involved in the terminal transfer of electrons from the quinone pool into the terminal components of the Nrf pathway and a formate-dependent nitrite reductase. Table S2. General features and genome sequencing information for [file pone.0315093.s001.pdf]

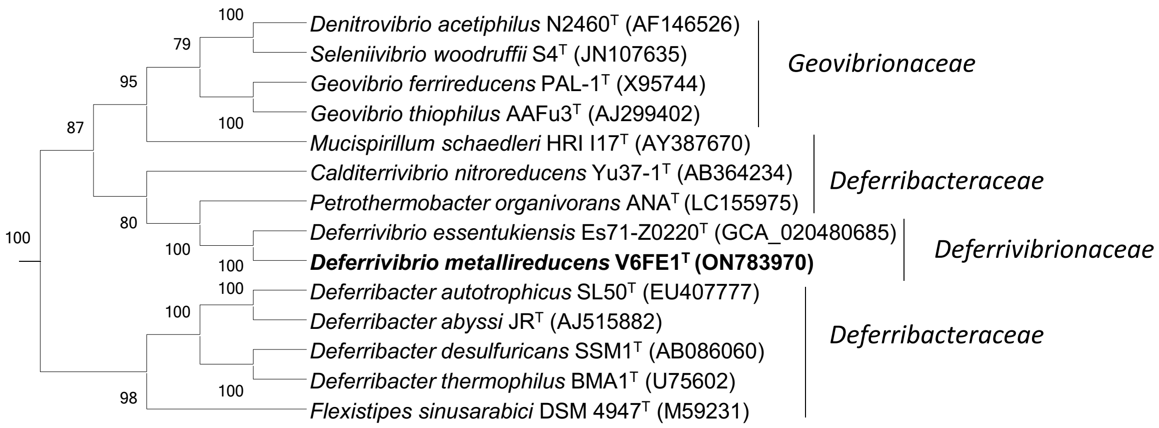

Figure S1. Cladogram based on 16S rRNA gene sequences showing the position of *Deferrivibrio metallireducens* V6FE1^{T} (ON783970) in the family *Deferrivibrionaceae,* related to the families *Deferribacteraceae* and *Geovibrionaceae* (Order *Deferribacterales*, Class *Deferribacteres*, Phylum *Deferribacterota*). The evolutionary history was inferred using the Maximum Likelihood method and General Time Reversible model. The percentage of trees in which the associated taxa clustered is shown next to the branches. This analysis involved 23 nucleotide sequences. The outgroup was composed of the following type species: *Thermanaeromonas toyohensis* ToBE (AB062280), *Moorella humiferrea* 64 FGQ (GQ872425), *Desulfovibrio indonesiensis* Ind 1 (Y09504), *Desulfovibrio marinus* E-2 (DQ365924), *Desulfonatronum cooperativum* Z-7999 (AY725424), *Desulfonatronum thioautotrophicum* ASO4-1 (FJ469577), *Desulfonatronum thiodismutans* MLF1 (AF373920), *Desulfonatronum lacustre* DSM 10312 (AF418171), *Desulfonatronum buryatense* Ki5 (KC417374). There were a total of 2753 positions in the final dataset. Evolutionary analyses were conducted in MEGA 7 (35). Taxonomy is based on LPSN.

DSMZ Identification Services polar lipid report

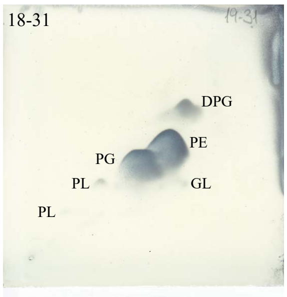

GL = Glycolipid

PL = Phospholipid

PG = Phosphatidylglycerol

PE = Phosphatidylethanolamine

DPG = Diphosphatidylglycerol

Figure S2. Polar lipids of strain V6Fe1^{T}. The polar lipid profile of strain V6Fe1T consisted of glycolipids, phospholipids, phosphatidylglycerol, phosphatidylethanolamine, and diphosphatidylglycerol

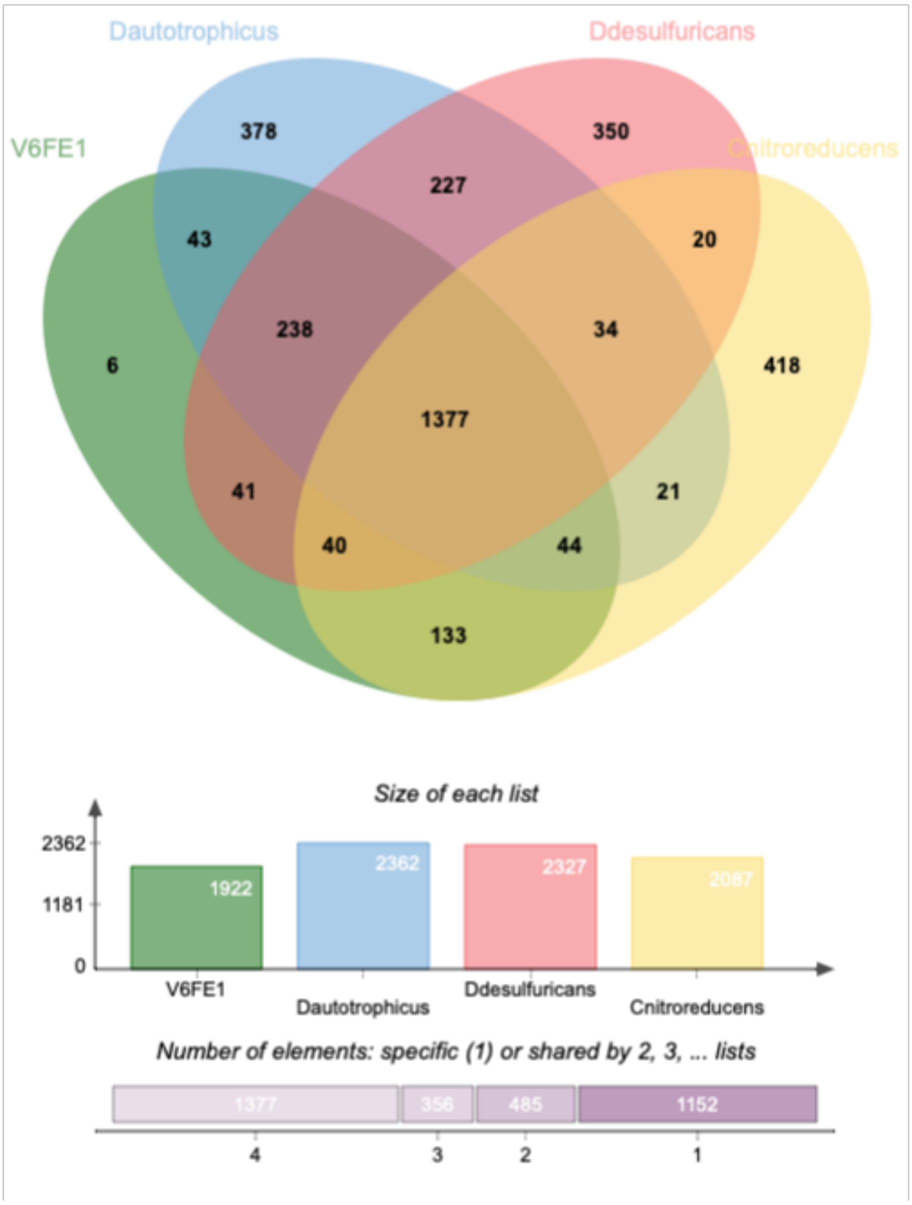

Figure S3. Venn diagram of protein orthologs shared by the assembly of *D. autotrophicus* chromosome and plasmid of *D. desulfuricans* and *C. nitroreducens*. Orthology analysis was performed with OrthoVenn2 web server using 0.01 blast e-value and 1.5 orthoMCL grain value. Numbers indicate shared or unique protein clusters and singleton proteins.

Table S1. Primers used in this study, designed using Primer3 software version 4.1.0, targeting *cym*A, *hcp, nap*A, *nor*V, *nrf*D (present in two copies), *nrf*A genes encoding respectively a cytoplasmic membrane electron transport protein involved in nitrate reduction, a hydroxylamine reductase, a periplasmic nitrate reductase, a nitric oxide reductase, an integral transmembrane protein involved in the terminal transfer of electrons from the quinone pool into the terminal components of the Nrf pathway and a formate-dependent nitrite reductase.

Gene name	Left primer 5’-> 3’	Right primer 5’-> 3’	Amplicon size
*cym*A	TAAGGAGTATGCAGCCGAGC	GCAAGAATATCCGGTCTGCC	192
Hcp	CTGTATTGTCCCGCCAAAGG	GTCCGCCCAAATCTTTAGCC	192
*nap*A	TGGTTTGCTACAAGTGGTGC	GCTGCCAGTTTCCCTTACAC	159
*nrf*A	ATTTGGCTGACGGTTCATGG	ACTCTTCACACTGGATGCCA	208
*nrf*D (direct)	AAGGACCTGCTAATCTGCCT	GAATACTGTTTCAAACAACC	184
*nrf*D (reverse)	CGTGTTAAGCCAAACCAGA	TGCCGTGATGCCTGAGATTA	152
*nor*V	CGGGTGCGCTTCCTAAAATT	TGTCAGGCCAGTGAAGGAAA	177

Table S2. General features and genome sequencing information for *Deferrivibrio metallireducens* V6Fe1^T according to MIGS recommendations.

**Investigation**	
Strain	*Deferrivibrio metallireducens* V6Fe1^T
Submitted to INSDC	SAMN 16483974
Project name	
Geographic location (latitude and longitude)	38,41° N, 14,96° E
Geographic location (country and/or sea, region)	Vulcano Island (Italy), near the seashore
Collection date	2015
Environnement (biome):	marine hydrothermal vent biome ENVO:01000030
Environnement (features) :	marine hydrothermal vent ENVO:01000122
Environnement (material) :	marine sediments ENVO:01001821
Depth :	50 cm
**General features**	
Classification	Domaine *Bacteria*
	Phylum *Deferribacteres*
	Classe *Deferribacteres*
	Ordre *Deferribacterales*
	Famille *Deferrivibrionaceae*
	Genus *Deferrivibrio*
	Species *metallireducens* (proposed)
Gram stain	Negative
Cell shape	Thin slightly curved rods
Motility	non motile
Growth temperature	45-65°C (optimum 55-60°C)
Relationship to oxygen	Aerotolerant (-> 10% O_2 in gaseous phase)
Trophic level	Chimiolithotrophic/chimioorganotrophic
Biotic relationship	Free-living
Isolation and growth conditions	This article
**Sequencing**	
Sequencing technology	Nanopore (long reads) MiSeq (short pair-ends reads)
Sequencing platform	Genotoul (Toulouse, France)
Assembler	MinKNOW ONT software (1.10.24-1 version)
Contigs number	0
Genome coverage	98.23 %
Genome assemblage NCBI	
Assembly	Contigs
**Genome features**	
Genome size (bp)	2,358,333
GC content (mol %)	34,83
CDS	2348
Number of RNAs	50
tRNAs	41
16S-23S-5S rRNAs	2-2-2
