# Supplementary material for: Metabolic versatility and nitrate reduction pathways of a new thermophilic bacterium of the Deferrivibrionaceae: Deferrivibrio metallireducens sp. nov isolated from hot sediments of Vulcano Island, Italy
Source: PLoS One. 2025 Mar 11;20(3):e0315093. doi: 10.1371/journal.pone.0315093 (PMC11896075; doi:10.1371/journal.pone.0315093)
Supplement: Attachment — Submitted filename: Reviewd 9.9.2024.pdf [file pone.0315093.s002.pdf]

PLOS ONE

Metabolic versatility and nitrate reduction pathways of a new thermophilic bacterium of the Deferrivibrionaceae: Deferrivibrio metallireducens sp. nov isolated from hot sediments of Vulcano Island, Italy

--Manuscript Draft--

**Manuscript Number:**	PONE-D-24-31234
**Article Type:**	Research Article
**Full Title:**	Metabolic versatility and nitrate reduction pathways of a new thermophilic bacterium of the Deferrivibrionaceae: Deferrivibrio metallireducens sp. nov isolated from hot sediments of Vulcano Island, Italy
**Short Title:**	Description of a new thermophilic bacterium and nitrate reduction pathways characterization.
**Corresponding Author:**	Grégoire GALES<br>Universite Aix-Marseille III: Aix-Marseille Universite<br>Marseille, FRANCE
**Keywords:**	Hydrothermalism; thermophilic; Deferrivibrionaceae; metabolic versatility; nitrate reduction pathways; Deferrivibrio metallireducens
**Abstract:**	A novel thermophilic anaerobic bacterium, designated strain V6Fe1T, was isolated from sediments affected by hydrothermal circulations of the Aeolian islands (Vulcano, Italy) on the seashore. Cells are thin, non-motile, Gram-negative flexible rods. Strain V6Fe1T grows at temperatures between 45-65 °C (optimum 55-60 °C), pH 5.1-7.7 (optimum 6.3-6.5), and NaCl concentration between 0-80 g/L (optimum 25 g/L). 16S rRNA gene sequence shows that Deferrivibrio essentukiensis, Petrothermobacter organivorans, Calditerrivibrio nitroreducens, and Deferribacter desulfuricans are the closest relatives (99.5%, 94.2%, 89.4%, and 88.8% 16S rRNA gene identity respectively). Strain V6Fe1T belongs to the recently described Deferrivibrionaceae family in the phylum Deferribacterota. Strain V6Fe1T showed substantial metabolic versatility. It grows chemoorganotrophically by fermenting proteinaceous substrates and organic acids or respiring organic compounds as electron donors and fumarate, nitrate, Fe(III), S°, and Mn(IV) as electron acceptors. Strain V6Fe1T grows chemolithoautotrophically using H2 as an electron donor and nitrate, nitrous oxide, Fe(III), Mn(IV), or sulfur as electron acceptors. Based on genomic (ANI) and phenotypic properties, strain V6Fe1T (= DSM 27501T = JCM 39088T) is proposed to be the type strain of a novel species named Deferrivibrio metallireducens.<br>Strain V6Fe1T is the only strain displaying denitrification within the Deferribacterota phylum. Dissimilatory nitrate reduction pathways were investigated with a stable isotope probing approach, showing that V6Fe1T performs both denitrification, nitrate being reduced to dinitrogen, and Dissimilatory Nitrate Reduction to Ammonium (DNRA). Culture experiments with nitrate as the sole electron acceptor and fumarate or H2 as electron donors were followed by quantitative RT-PCR and showed that strain V6Fe1T performs DNRA with the nitrite reductase formate-dependent NrfA and denitrification with an Hcp protein and other redox partners yet to be identified, Genome analysis and experimental data suggest that strain V6Fe1T performs autotrophic carbon fixation via the recently discovered reversed oxidative TCA cycle (roTCA cycle).
**Order of Authors:**	Grégoire GALES
	Mélanie Hennart
	Hannoun Maverick
	Postec Anne
	Erauso Gaël
**Additional Information:**	
**Question**	**Response**

Powered by Editorial Manager® and ProduXion Manager® from Aries Systems Corporation

**Financial Disclosure**<br><br>Enter a financial disclosure statement that describes the sources of funding for the work included in this submission. Review the submission guidelines for detailed requirements. View published research articles from *PLOS ONE* for specific examples.<br><br>This statement is required for submission and **will appear in the published article** if the submission is accepted. Please make sure it is accurate.<br><br>**Funded studies**<br>Enter a statement with the following details:<br>• Initials of the authors who received each award<br>• Grant numbers awarded to each author<br>• The full name of each funder<br>• URL of each funder website<br>• Did the sponsors or funders play any role in the study design, data collection and analysis, decision to publish, or preparation of the manuscript?<br><br>Did you receive funding for this work?	Yes
Please add funding details.<br>as follow-up to "**Financial Disclosure**<br><br>Enter a financial disclosure statement that describes the sources of funding for the work included in this submission. Review the submission guidelines for detailed requirements. View published research articles from *PLOS ONE* for specific examples.<br><br>This statement is required for submission and **will appear in the published article** if the submission is accepted. Please make sure it is accurate.	The project X-TREMOPHILES (coordinator G. Erauso, 2017-2019) was founded by the program X-Life of the Interdisciplinarity Mission (MITI) of the CNRS (France).

**Funded studies**<br>Enter a statement with the following details:<br>• Initials of the authors who received each award<br>• Grant numbers awarded to each author<br>• The full name of each funder<br>• URL of each funder website<br>• Did the sponsors or funders play any role in the study design, data collection and analysis, decision to publish, or preparation of the manuscript?<br><br>Did you receive funding for this work?"	
Please select the country of your main research funder (please select carefully as in some cases this is used in fee calculation).<br>as follow-up to "**Financial Disclosure**<br><br>Enter a financial disclosure statement that describes the sources of funding for the work included in this submission. Review the submission guidelines for detailed requirements. View published research articles from *PLOS ONE* for specific examples.<br><br>This statement is required for submission and **will appear in the published article** if the submission is accepted. Please make sure it is accurate.<br><br>**Funded studies**<br>Enter a statement with the following details:<br>• Initials of the authors who received each award<br>• Grant numbers awarded to each author<br>• The full name of each funder<br>• URL of each funder website<br>• Did the sponsors or funders play any role in the study design, data collection and analysis, decision to publish, or preparation of the manuscript?<br><br>Did you receive funding for this work?"	FRANCE - FR
**Competing Interests**	The authors have declared that no competing interests exist.

Use the instructions below to enter a competing interest statement for this submission. On behalf of all authors, disclose any competing interests that could be perceived to bias this work—acknowledging all financial support and any other relevant financial or non-financial competing interests.<br><br>This statement is required for submission and **will appear in the published article** if the submission is accepted. Please make sure it is accurate and that any funding sources listed in your Funding Information later in the submission form are also declared in your Financial Disclosure statement.<br><br>View published research articles from *PLOS ONE* for specific examples.<br><br>**NO authors have competing interests**<br><br>Enter: *The authors have declared that no competing interests exist.*<br><br>**Authors with competing interests**<br><br>Enter competing interest details beginning with this statement:<br><br>*I have read the journal's policy and the authors of this manuscript have the following competing interests: [insert competing interests here]*<br><br>* typeset	
**Ethics Statement**<br><br>Enter an ethics statement for this submission. This statement is required if the study involved:<br><br>• Human participants<br>• Human specimens or tissue<br>• Vertebrate animals or cephalopods<br>• Vertebrate embryos or tissues	N/A

• Field research<br><br>Write "N/A" if the submission does not require an ethics statement.<br><br>General guidance is provided below. Consult the submission guidelines for detailed instructions. **Make sure that all information entered here is included in the Methods section of the manuscript.**	

**Format for specific study types**<br><br>**Human Subject Research (involving human participants and/or tissue)**<br>• Give the name of the institutional review board or ethics committee that approved the study<br>• Include the approval number and/or a statement indicating approval of this research<br>• Indicate the form of consent obtained (written/oral) or the reason that consent was not obtained (e.g. the data were analyzed anonymously)<br><br>**Animal Research (involving vertebrate animals, embryos or tissues)**<br>• Provide the name of the Institutional Animal Care and Use Committee (IACUC) or other relevant ethics board that reviewed the study protocol, and indicate whether they approved this research or granted a formal waiver of ethical approval<br>• Include an approval number if one was obtained<br>• If the study involved *non-human primates*, add *additional details* about animal welfare and steps taken to ameliorate suffering<br>• If anesthesia, euthanasia, or any kind of animal sacrifice is part of the study, include briefly which substances and/or methods were applied<br><br>**Field Research**<br><br>Include the following details if this study involves the collection of plant, animal, or other materials from a natural setting:<br>• Field permit number<br>• Name of the institution or relevant body that granted permission	
**Data Availability**<br><br>Authors are required to make all data underlying the findings described fully available, without restriction, and from the time of publication. PLOS allows rare exceptions to address legal and ethical concerns. See the PLOS Data Policy and FAQ for detailed information.	Yes - all data are fully available without restriction

A Data Availability Statement describing where the data can be found is required at submission. Your answers to this question constitute the Data Availability Statement and **will be published in the article**, if accepted.<br><br>**Important:** Stating 'data available on request from the author' is not sufficient. If your data are only available upon request, select 'No' for the first question and explain your exceptional situation in the text box.<br><br>Do the authors confirm that all data underlying the findings described in their manuscript are fully available without restriction?	
**Describe where the data may be found in full sentences. If you are copying our sample text, replace any instances of XXX with the appropriate details.**<br><br>• If the data are **held or will be held in a public repository**, include URLs, accession numbers or DOIs. If this information will only be available after acceptance, indicate this by ticking the box below. For example: *All XXX files are available from the XXX database (accession number(s) XXX, XXX.)*.<br>• If the data are all contained **within the manuscript and/or Supporting Information files**, enter the following: *All relevant data are within the manuscript and its Supporting Information files.*<br>• If neither of these applies but you are able to provide **details of access elsewhere**, with or without limitations, please do so. For example:<br><br>*Data cannot be shared publicly because of [XXX]. Data are available from the XXX Institutional Data Access / Ethics Committee (contact via XXX) for researchers who meet the criteria for access to confidential data.*<br><br>*The data underlying the results presented in the study are available from (include the name of the third party*	The GenBank/EMBL/DDBJ accession number for the complete genome sequence of strain V6Fe1T is CP063375.

and contact information or URL).<br>• This text is appropriate if the data are owned by a third party and authors do not have permission to share the data.<br><br>* typeset	
Additional data availability information:	Tick here if the URLs/accession numbers/DOIs will be available only after acceptance of the manuscript for publication so that we can ensure their inclusion before publication.

Powered by Editorial Manager® and ProduXion Manager® from Aries Systems Corporation

Manuscript Click here to access/download;Manuscript;Article V6Fe1_29_juillet_2024_PlosOne.docx

Metabolic versatility and nitrate reduction pathways of a new thermophilic bacterium of the *Deferrivibrionaceae: Deferrivibrio metallireducens* sp. nov isolated from hot sediments of Vulcano Island, Italy

*Galès Grégoire; Mélanie Hennart, Hannoun Maverick, Postec Anne and *Erauso Gaël

[1] Aix-Marseille Université, Université du Sud Toulon-Var, CNRS/INSU, IRD, MIO, UM 110, 13288, Marseille, cedex 09

*Corresponding author: Grégoire Galès, gregoire.gales@univ-amu.fr or Gaël Erauso gael.erauso@univ-amu.fr

The GenBank/EMBL/DDBJ accession number for the 16S rRNA gene sequence of strain V6Fe1^T is ON783970.

The GenBank/EMBL/DDBJ accession number for the complete genome sequence of strain V6Fe1^T is CP063375.

Abstract

A novel thermophilic anaerobic bacterium, designated strain V6Fe1^T, was isolated from sediments affected by hydrothermal circulations of the Aeolian islands (Vulcano, Italy) on the seashore. Cells are thin, non-motile, Gram-negative flexible rods. Strain V6Fe1^T grows at temperatures between 45-65 °C (optimum 55-60 °C), pH 5.1-7.7 (optimum 6.3-6.5), and NaCl concentration between 0-80 g/L (optimum 25 g/L). 16S rRNA gene sequence shows that *Deferrivibrio essentukiensis*, *Petrothermobacter organivorans*, *Calditerrivibrio nitroreducens*, and *Deferribacter desulfuricans* are the closest relatives (99.5%, 94.2 %, 89.4 %, and 88.8 % 16S rRNA gene identity respectively). Strain V6Fe1^T belongs to the recently described *Deferrivibrionaceae* family in the phylum *Deferribacterota*. Strain V6Fe1^T showed substantial metabolic versatility. It grows chemoorganotrophically by fermenting proteinaceous substrates and organic acids or respiring organic compounds as electron donors and fumarate, nitrate, Fe(III), S°, and Mn(IV) as electron acceptors. Strain V6Fe1^T grows

chemolithoautotrophically using H_2 as an electron donor and nitrate, nitrous oxide, Fe(III), Mn(IV), or sulfur as electron acceptors. Based on genomic (ANI) and phenotypic properties, strain V6Fe1^T (= DSM 27501^T = JCM 39088^T) is proposed to be the type strain of a novel species named *Deferrivibrio metallireducens*.

Remove paragraph

Strain V6Fe1^T is the only strain displaying denitrification within the *Deferribacterota* phylum. Dissimilatory nitrate reduction pathways were investigated with a stable isotope probing approach, showing that V6Fe1^T performs both denitrification, nitrate being reduced to dinitrogen, and Dissimilatory Nitrate Reduction to Ammonium (DNRA). Culture experiments with nitrate as the sole electron acceptor and fumarate or H2 as electron donors were followed by quantitative RT-PCR and showed that strain V6Fe1T performs DNRA with the nitrite reductase formate-dependent NrfA and denitrification with an Hcp protein and other redox partners yet to be identified, Genome analysis and experimental data suggest that strain V6Fe1^T performs autotrophic carbon fixation *via* the recently discovered reversed oxidative TCA cycle (roTCA cycle).

Keywords: Hydrothermalism, thermophilic, *Deferrivibrionaceae*, metabolic versatility, nitrate reduction pathways, *Deferrivibrio metallireducens*

1. Introduction

Vulcano is the southernmost of the seven islands forming the Aeolian archipelago. This island harbors a developed shallow hydrothermal system. It is where the first hyperthermophilic marine archaeon, *Pyrodictium occultum,* was isolated (1), leading to extensive studies of the geochemical and microbiological properties of this environment (2)(3)(4). Several thermophilic and hyperthermophilic microorganisms have been isolated from Vulcano, including members of the *Bacteria* like *Aquifex aeolicus* (5) and *Thermotoga maritima* (6), members of the *Archaea* like *Archaeoglobus fulgidus* (7), *Ferroglobus placidus* (8), *Palaeococcus hegelsonii* (9), *Thermococcus celer* (10), *Pyrococcus furiosus* (11), *Pyrodictium occultum* [1], and *Staphylothermus marinus* (11).

Nitrate reducers are commonly found within hydrothermal systems (12)(13)(14). Thermodynamical calculation shows that nitrate may be one of the most suitable terminal electron acceptors for anaerobic respiration by chemolithotrophic or chemoorganotrophic lifestyles in Vulcano hydrothermal systems (12). The energy yield of nitrate reduction was shown to be comparable to that of oxidation reaction with O_2. Two microbial metabolic pathways for nitrate reduction are known: denitrification, meaning that nitrate is reduced to N_2 or some intermediates like NO or N_2O, or Dissimilatory Nitrate Reduction to Ammonium (DNRA) in which nitrate is reduced to ammonium. Most organisms rely on reactive forms of nitrogen for growth, such as ammonium and nitrate, as they cannot fix atmospheric or dissolved N_2. Reactive forms of nitrogen are often growth-limiting nutrients in environmental conditions (15). The fate of nitrate after being reduced is then critical, as ammonium produced during DNRA is still available in the environment, whereas N_2 is not. These processes are also of climate concern, as denitrification substantially releases the greenhouse intermediate N_2O (16). Nitrate reducers isolated from oceanic hydrothermal systems usually reduce nitrate to ammonium using sulfide or Fe(II) as an electron donor, like *Thermosulfurimonas dismutans* (17), *Dissulfuribacter thermophilus* (18), most of the *Zetaproteobacteria* (19), *Caldithrix abyssi* (20), *Nautilia nitratireducens* (21), *Thermosulfuriphilus ammonigenes* (22). Others reduce nitrate to nitrite, like *Vulcanibacillus modesticaldus* (23). A few nitrate reducers isolated from these environments perform complete denitrification, like *Sulfurimonas paralvinellae* (24), *Nitratiruptor tergarcus*, and *Nitratifractor salsuginis* (25), which belong to the *Epsilonproteobacteria*. Nitrate reduction metabolism has been investigated in Filamentous Large Sulfide-Oxidizing Bacteria (FLSB) of deep hydrothermal systems like the Guaymas basin (13). They were shown to be able to reduce their intracellular nitrate stocks to nitrogen gas and ammonium, depending on the concentration of electron donor (sulfide), by using stable isotope probing experiments and genome analysis (13).

Here, we describe a novel thermophilic strain V6Fe1^T, isolated from the Vulcano hydrothermal system, belonging to the *Deferribacterota* phylum and the recently described *Deferrivibrionaceae* family, closely related to the *Deferribacteraceae* family. Cultivated representatives of the *Deferribacteraceae* and *Deferrivibrionaceae* families are thermophilic anaerobes. Most *Deferribacteraceae* use electron acceptors such as ferric iron and nitrate. Fermentative growth within

Deferribacteraceae is rare and restricted to *D. desulfuricans* (26) and *P. organivorans* (27). Autotrophic growth occurs in *D. autotrophicus* (28) and *D. abyssi* (29). Strain V6Fe1^T shows a unique metabolic versatility within *Deferribacteraceae* since it ferments various organic substrates, performs anaerobic respiration using various electron acceptors (ferric iron, manganese, elemental sulfur, nitrate), and grows lithoautotrophically with H_2 as an electron donor. Contrary to its closest relatives, strain V6Fe1^T performs denitrification and DNRA, depending on the nature of the electron donor.

2. Materials and Methods

2.1 Geological setting and sampling methods

Sediment samples were collected from shallow hydrothermal marine vents on the shore of Vulcano Island at the Baia di Levante location (GPS coordinates 38°24'45'' N, 14°57' 38'' E). These sediments and the surrounding fluid were collected in 100 mL Schott bottles filled to the top and hermetically closed with a rubber stopper (no gas phase). They were stored at 4-8°C in the dark until used for culture.

2.2 Isolation of strain V6Fe1^T

For enrichment and isolation cultures, the Vulcano High Salt (VHS) medium was used, containing (per liter of distilled water): 18 g NaCl, 1 g $MgCl_2.6H_2O$, 1 g $MgSO_4.7H_2O$, 1 g NH_4Cl, 1 g $(NH_4)_2SO_4$, 0.3 g KCl, 1 g Na_2SO_4, 0.1 g $CaCl_2.2H_2O$, 0.5 g cysteine-HCl, 3.3 g PIPES, 0.1 g yeast extract, 1 ml K_2HPO_4/KH_2PO_4 1% solution, 1 ml trace mineral element solution (30), 1 ml Se-Ni-W solution (100 ml^{-1}: 10 mg Na_2SeO_3, 20 mg $NiCl_2.6H_2O$, 30 mg $Na_2WO_4.2H_2O$) and 1 ml of 0.1 % (w/v) resazurin (Sigma). The pH was adjusted to 6.3 with a 10 M KOH solution. The medium was boiled under a stream of O_2-free N_2 gas and cooled at room temperature. 50 ml or 5 ml of VHS medium were dispensed into penicillin flasks or Hungate tubes, respectively, degassed under N_2/CO_2 (80/20, v/v), and subsequently sterilized by autoclaving at 120 °C for 21 min. The Hungate technique (31) was used throughout the study. Sediments at 5 % w/v were first used as inoculum in 50 ml flasks supplemented with 13 mM poorly crystalline $Fe(OH)_3$ under 2 bars of H_2/CO_2 (80/20, v/v), then incubated at 60°C. Microbial growth was observed after three days of incubation and was accompanied by a change in the color of the medium, from rust-colored to dark brown, due to a reduction of ferric ions in ferrous ions. The enrichment culture was subcultured several times under the same growth conditions prior to isolation to

obtain pure cultures. The culture was then serially diluted tenfold in the same culture medium and solidified with 0.8 % Phytagel (Sigma) (w/v) using the roll tubes technique. The process of serial dilution in roll tubes was repeated three times until the isolates were deemed axenic. One strain, V6Fe1^T, was selected for further taxonomic and physiological characterization.

2.3 Taxonomic characterization and genome sequencing, assembly, and annotation

Extraction and purification of the genomic DNA from strain V6Fe1^T, PCR amplification, and sequencing of the 16S rRNA gene were performed as described previously (32). The sequence was compared with available sequences in GenBank using a BLAST search (33). The complete 16S rRNA gene sequence (1538 nucleotides) was later obtained from the genome sequence and deposited in the GenBank database under accession number ON783970. Selected sequences were aligned using Muscle (34), and evolutionary distances were calculated using the Maximum Likelihood method and the General Time Reversible model. Support for internal nodes was assessed by the bootstrap analysis (2000 replicates). These analyses were conducted in MEGA7 (35).

For genome sequencing, high molecular weight DNA was prepared from a 500 mL culture (basal medium with 20 mM fumarate and 2 g/L yeast extract) cell pellet using a method based on a phenol-chloroform-isoamyl alcohol extraction as previously described (36). Genomic sequencing was performed at the GenoToul platform (Toulouse, France) by combining long reads technology of Oxford Nanopore to ease assembling (GridION) and high coverage provided by short paired-end reads obtained with Illumina (MiSeq) technology. The methodological details on sequencing, read processing and *de novo* assembly of the genome are provided in Supplementary Methods. Annotation of the genome was performed using the MicroScope platform (37). The complete genome sequence has been deposited under the accession number CP063375.

Digital hybridization values dDDH were calculated using the Genome-to-Genome Distance Calculator (GGDC) 2.1 (http://ggdc.dsmz.de/). Average nucleotide Identity (ANI) and Percentage Of Conserved Proteins (POCP) were computed using Protologger v0.9974 (http://www.protologger.de/) (38). The detection of orthologous proteins shared by strain V6Fe1^T, *D. autotrophicus, D. desulfuricans,* and *C. nitroreducens* was performed by the OrthoVenn2 web server (39). Operons, promoters, and

terminators predictions were performed by using the FgenesB, BPROM, and FindTerm online tools at (http://www.softberry.com/berry.phtml)

For 16S rRNA gene analysis, the sequences were extracted from genome assemblies using barrnap 0.9 (https://github.com/tseemann/barrnap) and the sequences were aligned with MAFFT 7.515 (40). The resulting alignment was used for phylogenetic tree inference with IQ-TREE 2.3.4 (41) using the GTR+F+G4 model. Branch support was obtained after 1000 bootstrap replicates.

For phylogenetic analysis based on 120 bacterial marker genes, the sequences were extracted and aligned from genome assemblies using GTDB-Tk 2.3.2 (42) with the GTDB Release 214 and phylogenetic tree inference using IQ-TREE 2.3.4 (41) with LG+I+R4 model. Branch support was obtained after 1000 bootstrap replicates.

2.4 Physiological and metabolic characterization

Growth experiments were performed in triplicate. pH, temperature, and NaCl concentration ranges for growth, as well as the oxygen, antibiotics, nitrite (2 mM), and sulfite (2 mM) sensitivities, were determined using the VHS medium supplemented with fumarate (20 mM) and yeast extract (2 g/L). When gas supply was required for metabolism studies (e.g., with H_2 or CO tested as electron donors, NO_2 tested as an electron acceptor, or when oxygen tolerance was tested), cultures were performed in 120 mL penicillin bottles containing 50 mL of medium, and incubated at 60°C with shaking at 120 rpm. To determine optimal growth temperature, water baths were used for incubating bacterial cultures from 20 °C to 75 °C, with increments of 5 °C. The pH of the medium was adjusted by injecting in Hungate

Used same formate for celcious

tubes aliquots of anaerobic stock solutions of 1 M HCl (acidic pHs), 10 % $NaHCO_3$, or 8 % Na_2CO_3 (alkaline pHs) to test pH between 4.0 and 8.6 and checked after autoclaving. To study the salinity requirement, NaCl was weighed directly in the tubes (0-10 %, w/v) before the medium was dispensed therein. Strain V6Fe1^T was subcultured twice under the same experimental conditions before the growth rates were determined. The culture conditions were then fixed with the optimal temperature, pH, and salinity parameters determined from these experiments.

The oxygen sensitivity of strain V6Fe1^T was tested at 60°C, up to 12.5% O_2 in the gas phase in 50 mL vials with a 70 mL headspace under agitation (120 rpm). Susceptibility to antibiotics was tested twice with a final concentration of 100 µg/l of the following antibiotic injected from sterile, anaerobic

stock solutions and tested separately: ampicillin, bacitracin, chloramphenicol, cycloheximide, gentamicin, kanamycin, streptomycin, tetracycline, and vancomycin as tested in (27).

To determine which substrates were used by strain V6Fe1^T, Balch vitamins (30) were added in all experiments without proteinaceous substrates except for testing autotrophy. The headspace consisted of 100% N_2 except when testing autotrophic growth, where the gas phase was H_2/CO_2 (80/20, v/v 2 bar). When testing autotrophic capabilities, cysteine and PIPES were omitted in the medium. Carbon monoxide (CO) in the gas phase (N_2/CO 90/10% vol/vol) was tested as an electron donor using nitrate as the electron acceptor (43). To ensure the capacity of strain V6Fe1^T to reduce nitrous oxide to dinitrogen, strain V6Fe1^T was cultured with nitrous oxide as the sole electron acceptor in the medium, with H_2, glucose, or acetate. Gaseous phases were fixed at $N_2O/H_2/CO_2$ (33/54/13 vol/vol 1 bar) or $N_2O/N_2/CO_2$ (33/54/13 vol/vol 1 bar) according to (44), for testing nitrous oxide utilization with H_2 or acetate (20 mM) respectively as an electron donor. The vials were incubated with shaking at 120 rpm at 60 °C.

Sugars (arabinose, cellobiose, fructose, galactose, glucose, N-acetyl glucosamine, glycerol, lactose, maltose, mannose, raffinose, ribose, rhamnose, saccharose, sorbose, xylose), polymers of sugars (starch), alcohols (ethanol, mannitol, methanol), proteinaceous substrates (casamino of acids, peptone, tryptone, yeast extract) and organic acids (acetate, butyrate, citrate, crotonate, formate, fumarate, lactate, malate, propionate, pyruvate and succinate) were tested as growth substrates for strain V6Fe1^T. Each substrate was added to the VHS medium from sterile anaerobic stock solutions to a final concentration of 20 mM, formate to 100 mM, and starch was added before autoclaving at a final concentration of 10 g/L.

Elemental sulfur (0.5 % w/v), sodium sulfate (20 mM), sodium thiosulfate (20 mM), sodium sulfite (2 mM), fumarate (20 mM), sodium nitrate (10 mM), sodium nitrite (2 mM), manganese oxide MnO_2 (13 mM) and iron oxide as poorly crystalline $Fe(OH)_3$ (13 mM), goethite (13 mM) or soluble forms such as ferric citrate or Fe(III)-EDTA (13 mM each) were tested each as terminal electron acceptors in the presence of the following electron donors tested separately at concentrations already mentioned: arabinose, cellobiose, fructose, galactose, glucose, glycerol, lactose, maltose, mannose, raffinose, ribose, rhamnose, saccharose, sorbose, xylose, starch, ethanol, mannitol, methanol, casamino of acids, peptone,

tryptone, yeast extract, acetate, butyrate, citrate, crotonate, formate, fumarate, lactate, malate, propionate, pyruvate and succinate, H_2/CO_2 80/20 v/v at 2 bar as electron donors. When elemental sulfur was provided, autoclaving was performed at 105°C for one hour to prevent sulfur melting. Nitrogen-fixing capabilities were determined in the VHS medium without ammonium supplemented with 20 mM fumarate under N_2/CO_2 (80/20, v/v 1 bar). Bacterial growth was monitored by microscopic observation and counting using a Helber Bacteria Counting Chamber at 0.02 mm depth (Hawksley, England).

2.5 Cell morphology and chemotaxonomy

Cell morphology and purity of the strains were assessed under an Optiphot (Nikon) phase contrast microscope. For ultrastructure studies, cells were negatively stained with sodium phosphotungstate, as previously described (45) and observed under a Zeiss EM912 microscope. The presence of spores was checked by microscopic observation of cultures and after pasteurization tests performed at 80, 90, and 100 °C for 10 and 20 min and in old (more than three weeks) cultures. We tried to stimulate flagella production, providing a remote electron acceptor to the strain. To do that, motility assays were performed in anaerobic tubes by suspending fresh cultures of strain V6Fe1^T in VHS mineral medium with soft agar (0,1 to 1 %). After solidification, the cell suspension is covered with sterile VHS medium with soft agar (0,1 to 1 %) supplemented with 13 mM Fe(III) and 2 g/L yeast extract under N_2/CO_2 (80/20, v/v) or H_2/CO_2 (80/20, v/v) at 1 bar each.

Fatty acids were extracted using Miller's modified method (46) and analyzed by gas chromatography (model 6890N, Agilent Technologies) using the Microbial Identification System (MIDI, Sherlock Version 6.1; database, ANAER6). Analyses of the respiratory quinones and polar lipids were conducted at the Identification Service of the DSMZ (Braunschweig, Germany). The G+C content was determined from the genome sequence.

2.6 Chemical analyses

Sulfide production was determined *via* the adaptation of a protocol from the LCW 053 Hach kit. Ammonium production was measured spectrophotometrically using the indophenol blue method (47). Organic compounds were measured by HPLC (Dionex Ultimate 3000). Gas concentrations (N_2O, CO_2,

H_2, CO) were measured by gas chromatography (Shimadzu GC 8A). Nitrate, nitrite, sulfate and thiosulfate concentrations were measured by ion chromatography (Dionex Easion). The converting rates of nitrate to ammonium and/or nitrogen, fumarate to succinate and/or carbon dioxide by whole cells were calculated by comparing the rate of consumption of supplemented substrates and/or formation of the corresponding products.

2.7 Investigation of nitrate reduction metabolic pathways

The metabolic fate of ^{15}N-labeled nitrate was investigated to identify terminal products of nitrate reduction. Culture media containing $^{15}NO_3^-$ 2 mM (Aldrich) were prepared with or without 10 mM unlabeled nitrate as an electron acceptor in the presence of fumarate 15 mM or H_2/CO_2 (80/20, v/v) as electron donors. After one week of incubation at 60 °C, gaseous dinitrogen and dissolved ammonium isotopic ratio were measured by mass spectrometry (CN-Integra tracer-mass) according to (48). Briefly, 2 ml of final culture supernatant was treated in a stoppered flask with mild alkali (MgO); conversion of NH_4^+ to volatile NH_3 was carried out at 60°C for four days. Evolved NH_3 was collected as N-NH_4^+ in an acidified (coated with 0.5 M H_2SO_4) disk cut from a GF/D filter (Whatman) suspended on the stopper. Each disk was rolled into a pellet in the laboratory and fed into a mass spectrometer. An organic standard (glycine) was used to calibrate the measurements and track the Dumas combustion's consistency.

A quantitative RT-PCR approach was used to quantify the expression of several genes involved in nitrate reduction pathways. To determine which genes were expressed according to the electron donor furnished during nitrate reduction, strain V6Fe1^T was cultivated in three conditions: condition F with fumarate 15 mM as the only substrate (reference condition for gene expression), condition FN with 15 mM fumarate and 10 mM nitrate and condition HN with H_2/CO_2 (80/20, v/v) and 10 mM nitrate (autotrophic condition). Cultures of 100 mL in 300 mL glass flasks, hermetically closed with a rubber stopper and aluminum seal, were incubated at 60 °C, with shaking at 120 rpm. Aliquots of 2 mL were regularly sampled for chemical analyses and cell counting. When cells reached the mid-exponential phase, 4 mL of culture were sampled, centrifuged, and immediately stored at -80°C for later RNA extraction.

Specific primers for the selected genes (Sup. Table 1) were designed using Primer3 software version 4.1.0 and ordered to Eurogentec (Liège, Belgium). Automatic genome annotation allowed us to

identify the following genes: *cym*A DSN97_06310, *hcp* DSN97_01195, *napA* DSN97_05245, *nor*V DSN97_07985, *nrfD* (present in two adjacent reversed copies DSN97_01825 and DSN97_11315). They encode respectively a cytoplasmic membrane electron transport protein involved in nitrate reduction by *Shewanella oneidensis* (49), a hydroxylamine reductase, a periplasmic nitrate reductase, a nitric oxide reductase and an integral transmembrane protein involved in the terminal transfer of electrons from the quinone pool into the terminal components of the Nrf pathway. A blast search within the V6Fe1^T genome revealed the presence of a putative *nrfA* gene DSN97_03745, encoding for a pentaheme nitrite reductase, putatively involved in DNRA. Primers of 20 to 24 bp have been designed to generate amplicons of 152-208 bp length and a melting temperature between 60 and 62°C (Sup. Table 1), except primers targeting the 16S rRNA gene DGGE300F1 (50) and univ5161 (51), already available at the lab, whose sequence differed only by one base from those of strain V6Fe1^T.

RNA extractions were performed with the High Pure RNA Isolation Kit (Roche). RNA samples were then treated with the TurboDNaseTM (Ambion®, Thermo Fisher Scientific Corp.) according to the manufacturer's recommendations and reverse transcribed into cDNA using the SuperScript® IV Reverse Transcriptase (Invitrogen). The relative gene expression levels were quantified using SYBR Green on a CFX Real-Time PCR system (Biorad). The expression levels of all the genes were normalized to 16S rRNA gene expression. Gene expression was normalized against the 16S rRNA gene using the $2^{-\Delta CT}$ calculation: $\Delta CT = CT_{\text{gene of interest}} - CT_{\text{16S rRNA}}$ (CT, threshold cycle).

3 Results and discussion

3.1 Metabolic properties and taxonomic characterization of strain V6Fe1^T

Environmental samples were collected from shallow hydrothermal sediments in Baia di Levante, Vulcano Island, Italy. These were hydrothermally heated sediments, debris flow deposits, and poorly sorted coarse sands to fine gravels, with some millimetric leucite gravels corresponding to the high alkaline character of the lavas (52). The fluid temperature at the emission point was 55-65 °C and pH 6.25. Enrichment cultures were performed from these sediments, with an H_2/CO_2 (80/20, v/v) gas phase, $Fe(OH)_3$ 13 mM as the electron acceptor, and 0.1 g/L yeast extract. Brown coloration within three days indicated iron reduction. The Hungate technique was used throughout the isolation procedure. Several

black colonies with diameters ranging from 1.0 to 2.0 mm developed after 3-5 days of incubation at 60°C and were picked separately. After serial dilution on a solid medium, a new strain named V6Fe1^T was isolated and characterized. Based on its 16S rRNA gene sequence, strain V6Fe1^T belongs to the class *Deferribacteres*. The most closely related type species are *Deferrivibrio essentukiensis* Es71-Z0220^T (99.5 % 16S rRNA gene identity) (53), *P. organivorans* ANA^T (94.2 % identity) (27), *C. nitroreducens* YU37-1^T (89.4 % identity) (54) and *D. desulfuricans* SSM1^T (88.8 % identity) (26). A comparison of 1538 nucleotides of the 16S rRNA gene sequence of strain V6Fe1^T with those available in the GenBank database was used to infer a phylogenetic tree, which placed strain V6Fe1^T close to thermophilic bacteria belonging to the genera *Deferrivibrio*, *Petrothermobacter*, *Calditerrivibrio* and *Deferribacter* (Figure 1). A genomic tree (Figure 2) was also inferred from 120 bacterial marker genes by GTDB-Tk, showing an equivalent position of *Deferrivibrio metallireducens* V6Fe1^T in the family *Deferrivibrionaceae*, related to the families *Deferribacteraceae* and *Geovibrionaceae*, as a cladogram presented on supplementary Figure S1, based on 16S rRNA gene sequences, inferred using the Maximum Likelihood method and General Time Reversible model.

Cells of strain V6Fe1^T are non-motile and produce thin (0.2-0.8 µm x 4-8 µm) slightly curved rods. Cells of strain V6Fe1^T stain Gram-negative (Gram staining reaction). Indeed, ultrathin sections of cells observed by MET confirmed a Gram-negative type of cell wall (Fig. 3). No spore formation was observed in any tested condition, as is the case with all members of the *Deferribacterota* phylum. No flagella were observed. Results in motility tests and flagella observation are contrasted within the *Deferribacterota* phylum. *D. essentukiensis* (53), *C. nitroreducens* (54), *D. autotrophicus* (28), and *D. abyssi* (29) exhibit motility with a polar flagellum; *D. desulfuricans* (26) and *D. thermophilus* (55) exhibit a polar flagellum but no motility whereas *P. organivorans* exhibit no polar flagellum nor motility. Motility was not observed under phase contrast or transmission microscopy. Strain V6Fe1^T exhibited no polar flagellum in our culture conditions, whereas 40 genes involved in the flagellum synthesis (for example 8 genes in an operon DSN97_01525-60) and 12 genes involved in chemotaxis (for example CheR DSN97_07015, CheV DSN97_00765, CheW DSN97_06610, CheY DSN97_07060 and CheZ DSN97_07055) are found in the genome. No motility could be observed after sampling strain

V6Fe1^T from soft agar assays. The motility of strain V6Fe1^T could be of gliding type, but it has not been assessed for now.

Analysis of the lipid composition of strain V6Fe1^T membranes showed that the major fatty acids are anteiso-$C_{15:0}$ (22.2 %), iso-$C_{14:0}$ (13.1 %), $C_{16:0}$ (6.8 %), iso-$C_{17:0}$ (6.8 %), anteiso-$C_{17:0}$ (6.3 %), iso-$C_{16:0}$ (6.1 %), anteiso-$C_{17:1}$ (6.1 %), iso-$C_{17:1}$ (5.9 %), $C_{18:0}$ (3.7 %), $C_{17:0}$ (2.6 %), iso-$C_{16:1}$ (2.0 %), $C_{16:1}$ (1.7 %). Major fatty acids of strain V6Fe1^T differ from those of *D. essentukiensis*, as unsaturated fatty acids account for more than 15 % in strain V6Fe1^T, whereas unsaturated fatty acids of *D. essentukiensis* only account for 4 %. This discrepancy could be due to the different salinities in their environments (56). Strain V6Fe1^T inhabits a hydrothermal heated seawater environment, whereas *D. essentukiensis* inhabits mineral water deposits with low salinity (53), as *C. nitroreducens,* in whose cell wall no unsaturated fatty acid was detected, does (54). Indeed, unsaturated fatty acids of *Limisalsivibrio acetivorans* L21-Ace-BES^T, a recently discovered bacterium belonging to the *Deferribacteres* class, isolated from a hypersaline lake, account for 48,7% of the total fatty acid content (57). Major respiratory quinones are menaquinone MK-7 40% and MK-8 60%. MK-7 is thought to be the principal cofactor of CymA, a quinone-converting enzyme that plays a central role in the multibranched respiratory chain of *S. oneidensis* (58). The polar lipid profile of strain V6Fe1^T consisted of glycolipids, phospholipids, phosphatidylglycerol, phosphatidylethanolamine, and diphosphatidylglycerol (supplementary Figure S2).

The phenotypic characteristics of strain V6Fe1^T are listed in Table 1. It grows at temperatures between 45 °C and 65 °C, with an optimum at 60 °C (no growth at 40 °C and 70 °C). Growth occurred at NaCl concentrations between 0 and 80 g/L, with an optimum at 25 g/L (no growth at 90 g/L). Growth occurred between pH 5.1 and 7.7, with optimal growth at pH 6.3–6.5. In optimal fermentative conditions, e.g., with 20 mM fumarate and 2 g/L yeast extract, doubling time was measured at 120 minutes. Strain V6Fe1^T was resistant to streptomycin and vancomycin, while it was sensitive to ampicillin, bacitracin, chloramphenicol, cycloheximide, gentamicin, kanamycin, and tetracycline. Strain V6Fe1^T growth was inhibited by nitrite or sulfite (both at 2 mM). Strain V6Fe1^T showed an oxygen tolerance of up to 0.6% in the gas phase, while no growth was observed with 1.5 % oxygen in the gas

phase, as is the case for *D. thermophilus* (55). *D. essentukiensis* and *C. nitroreducens* did not tolerate 2% oxygen in the gas phase and were described as an obligate anaerobes (53)(54).

Fermentation is a rare trait within members of the *Deferribactota* phylum and has been observed only for *P. organivorans* (27) and *D. desulfuricans* (26). *D. essentukiensis*, the closest relative of strain V6Fe1^T, cannot ferment any substrate (53), like *C. nitroreducens* (54). Sugar utilization within members of the *Deferribacterota* phylum is also rare, as only *D. autotrophicus* can use maltose as a carbon and energy source (28). Strain V6Fe1^T can use the following organic acids and proteinaceous substrates as carbon and energy sources: butyrate, fumarate, lactate, malate, pyruvate, succinate, yeast extract, peptone, tryptone, casamino acids. Sugars (arabinose, cellobiose, fructose, galactose, glucose, N-acetyl glucosamine, lactose, mannose, rhamnose, ribose, saccharose, starch, xylose), propionate, citrate, methanol, ethanol, formate, and H_2/CO_2 (80/20, v/v) in the presence of acetate (2 mM acetate) were not used. Strain V6Fe1^T can grow by anaerobic respirations, using organic acids or proteinaceous substrates as electron donors, with Fe(III), Mn(IV), S° and NO_3^- as electron acceptor but not sulfate nor thiosulfate or soluble Fe(III) forms. Strain V6Fe1^T could not use carbon monoxide as an electron donor as *D. autotrophicus* (43). Indeed, the carbon monoxide dehydrogenase gene was not found in its genome. Yeast extract was not necessary for the growth of strain V6Fe1^T but strongly stimulated it.

Autotrophic growth was examined in a minimal VHS medium without organic carbon (i.e., without yeast extract, Balch vitamins, and PIPES salt). Strain V6Fe1T was able to grow under autotrophic conditions using CO_2 as the sole carbon source, H_2 as the sole energy source, and NO_3^-, N_2O, Fe(III), Mn(IV), or S° as electron acceptors.

Strain V6Fe1^T shared many phenotypic features with representatives of the genera *Deferrivibrio, Petrothermobacter, Calditerrivibrio,* and *Deferribacter* (Table 1). Species of these genera were mainly isolated from hydrothermal vents or terrestrial hot springs. In contrast, *D. essentukiensis* (53) was isolated from a subsurface aquifer, and *D. thermophilus* (55) and *P. organivorans* (27) were isolated from oil reservoirs. All these strains have a Gram-negative type of cell wall structure. Strains within the *Deferribacterales* order (*Deferrivibrio, Deferribacter, Calditerrivibrio,* and *Petrothermobacter* species) are rod-shaped and grow by anaerobic respiration (by oxidation of a variety of complex organic compounds and organic acids in the presence of diverse electron acceptors), but few can ferment. On

the contrary, strain V6Fe1^T and its close relatives *Petrothermobacter organivorans* and *D. desulfuricans* can grow by fermentation of proteinaceous compounds and organic acids. Within the *Deferribacterota* phylum, only *D. autotrophicus* (28), *D. abyssi* (29), and strain V6Fe1^T can grow chemolithoautotrophically using CO_2 as the sole carbon source and H_2 as the electron donor.

Table 1. Characteristics that differentiate strain V6Fe1^T from its closest relatives

Strains: 1, strain V6Fe1^T; 2, *Deferrivibrio essentukiensis* Es71-Z0220^T (53), 3, *Petrothermobacter organovorans* ANA^T (27); 4, *Calditerrivibrio nitroreducens* YU37-1^T (54); 5, *D. desulfurricans* SSM1^T (26); 6, *D. abyssi* (29); 7, *D. autotrophicus* SL50^T (28).

+, Positive; -, negative.

Characteristic	**1**	**2**	**3**	**4**	**5**	**6**	**7**
Temperature range (°C)	45-65	30-54	25-60	30-65	40-70	45-65	25-75
Optimum (°C)	60	47	55	55	60-65	60	60
NaCl range (%, w/v)	0-8	0-1.8	0-6	0-0.5	1.8-9.6	1-5	1-6
Optimum (%, w/v)	2.5	0.7	2.5	0.15	3.6	3	2.5
pH range	5.1-7.7	6.2-7.9	6-8	5.5-8	5-7.5	6-7.2	5-7.5
Optimum	6.3	7.1	7	7-7.5	6.5	6.5	6.5
Fermentation	+	-	+	-	+	-	-
Electron donors							
Ethanol	-	-	-	ND	+	-	-
Formate	+	-	ND	-	+	-	+
Propionate	-	ND	-	-	+	-	+
Fumarate	+	ND	+	+	+	ND	-
Electron acceptors							
Elemental sulfur	+	+	ND	-	+	+	+
Fe(III)	+	-	+	-	-	+	+
Mn(IV)	+	ND	+	-	-	-	+
Sulfate	-	-	+	-	-	-	-
Thiosulfate	-	+	ND	-	-	-	-
Nitrate	+	-	+	+	+	+	+
Tolerance to O_2	-		-	ND	-	-	-
End-product of nitrate reduction	NH_3/N_2	NH_3	NO_2^-	NH_3	NO_2^-	NO_2^-	NH_3
DNA G + C content (mol%)	34.8	34.04	34.3	35.1	38.6	30.8	28.7

Proper Table alignment

3.2 General genome properties

The genome of strain V6Fe1^{T} consists of a circular chromosome with an overall size of 2,358,333 bp and a G + C content of 34,83 mol %, similar to that of closely related strains with sequenced genomes (2,382,387 bp for *D. essentukiensis*, 2,216,552 bp for *C. nitroreducens* and 2,542,993 bp for *D. desulfuricans*). Strain V6Fe1^{T} has no plasmid, whereas *D. desulfuricans* harbors a 5.9 kb plasmid and *C. nitroreducens* a 58.7 kb plasmid.

General features of strain V6Fe1^{T} genome are listed in supplementary Table S2. The genome contains 2356 coding sequences (CDS), of which 2254 are protein coding. Of the 2254 protein-coding genes, 1937 were given a predicted function. Signal transduction mechanisms, ribosome structure and biosynthesis, membrane and envelope biosynthesis, and cell motility were among the most represented functional categories with respectively 7.5, 6.3, 5.8, and 4.2 % of the function-predicted genes. Strain V6Fe1^{T}, *D. essentukiensis, D. desulfuricans, D. autotrophicus* and *C. nitroreducens* are close relatives (Figure 1). *D. desulfuricans, D. autotrophicus* and *C. nitroreducens* share 1696, 1702 and 1594 proteins, respectively within 1922 strain V6Fe1^{T} proteins detected by the Orthovenn2 web server (Supplementary Figure S3).

Measurement of ANI values showed that strain V6Fe1^{T} shared 89.79 %, 76.91 %, and 76.89 % ANI with *D. essentukiensis, D. desulfuricans,* and *C. nitroreducens*, respectively, clearly indicating that strain V6Fe1^{T} belongs to a new species, given that the recommended threshold value for a new species is around 95-96 % (59). The dDDH values between the genomes of strains V6Fe1^{T} and *D. essentukiensis* was 77.8%, while they were much lower with *D. desulfuricans, C. nitroreducens, Flexistipes sinusarabicus,* and *D. autotrophicus* respectively 13.3%, 13.0%, 12.8%, and 12.6%. Percentage of Conserved Proteins (POCP) calculated with Protologger indicated values of 68.05 % with *D. desulfuricans,* 67.32 % with *C. nitroreducens,* 63.34 % with *F. sinusarabici,* 47.89 % with *Denitrovibrio acetiphilus* and 38.55 % with *Mucispirillum schaedleri*. A 50 % POPC value threshold was recommended to define a new genus (60). Such a recommendation should lead to transferring and reclassifying all *Deferrivibrio, Deferribacter, Calditerrivibrio, Flexistipes, and Petrothermobacter* species into the same genus. Classification of strain V6Fe1^{T} as a new species of the genus *Deferrivibrio* is proposed within the *Deferrivibrionaceae* family.

3.3 Central Carbon Metabolism

Experimental cultures showed that strain V6Fe1^T can oxidize various organic compounds (proteinaceous compounds and organic acids) for growth. Indeed, the genome of strain V6Fe1^T encodes glycoside hydrolases (of which one α-amylase), proteases and peptidases, alcohol and aldehyde dehydrogenases, and genes coding for ABC-transporters of sugars and peptides. Whereas sugars were not used by strain V6Fe1^T during our experiments, searches against the carbohydrates-active enzymes (CAZYmes) database (61) identified 47 CAZymes, including 11 glycoside hydrolase, 29 glycoside transferase, 2 carbohydrate esterase, and 5 carbohydrate-binding enzyme genes. These enzymes may be involved in the peptidoglycan metabolism. Sugars are generally not used by members of the *Deferribacterota,* except for *D. autotrophicus*, which can oxidize maltose with nitrate as an electron acceptor (28). Gluconeogenesis pathways, pentose phosphate pathway (only non-oxidative phase), and a complete Embden-Meyerhoff pathway were also identified. The genes for the complete biosynthesis pathways of many amino acids are present: alanine, arginine, aspartate, cysteine, glutamate, glutamine, glycine, phenylalanine, threonine, and tyrosine. The genome also encodes five dipeptides and at least 18 amino-acids ABC transporters with specificity for polar amino acids and branched amino acids, confirming amino acids as substrates for growth.

The presence of multiple cytochromes and ATP synthase subunits, as well as menaquinones genes, affords strain V6Fe1^T's respiratory capacities. This strain harbors a complete set of enzymes involved in the Krebs cycle. Oxidative phosphorylation in strain V6Fe1T is performed by a typical F0F1-ATP Synthase, whose subunits are encoded by two distinct operons (DSN97_00830-860 and DSN97_06300-305).

Strain V6Fe1^T grows chemolithoautotrophically using CO_2 as the carbon source and H_2 as an electron donor by anaerobic respiration of Fe(III), Mn(IV), S°, NO_3^-, and N_2O. Therefore, genes encoding the key enzymes of microbial carbon fixation pathways were searched in the V6Fe1^T genome. The following genes were not found: ribulose 1,5-bisphosphate carboxylase (Calvin-Benson cycle), carbon monoxide dehydrogenase/acetyl-CoA synthase complex (reductive acetyl-CoA pathway), ATP-citrate lyase and citryl-CoA lyase (two variants of the reductive tricarboxylic acid cycle), 4-hydroxybutyryl-CoA dehydratase (3-hydroxypropionate/4-hydroxybutyrate and dicarboxylate/4-

hydroxybutyrate cycles), and malonyl-CoA reductase (3-hydroxypropionate bi-cycle). We found that the V6Fe1^T genome encodes enzymes of the reductive citric acid cycle, including a fumarase (DSN97_05680), also present in the genome of *D. autotrophicus, D. desulfuricans, C. nitroreducens,* and *D. essentukiensis*. It also contained a citrate synthase gene (DSN97_09950), whose activity could be involved in carbon fixation. Recently, a new carbon fixation pathway has been proposed for the sulfur-reducing anaerobic deltaproteobacterium *Desulfurella acetivorans* (62). Under chemolithoautotrophic conditions, citrate synthase can cleave citrate adenosine triphosphate into acetyl coenzyme A and oxaloacetate. Therefore, the TCA cycle operates in the reductive direction with the reverse reaction of citrate synthase. Acetyl-CoA is reductively carboxylated to pyruvate by ferredoxin-dependent pyruvate synthase. The presence of genes of anaplerotic enzymes, pyruvate carboxylase, phosphoenolpyruvate carboxykinase, and oxaloacetate-decarboxylating malate dehydrogenase indicates the possibility of maintaining the level of the cycle intermediates. Considering all these observations, CO_2 fixation can occur in strain V6Fe1^T, most likely via the recently discovered roTCA cycle ("reversed oxidative TCA cycle") (62), as strain V6Fe1^T genome contains the same genes involved in carbon fixation as *Desulfurella activorans.*

The capacity for autotrophic growth was experimentally shown for *D. autotrophicus* (28) and *D. abyssi* (29) and suggested for *Geovibrio thiophilus* (it can be repeatedly subcultured with H_2/CO_2 and S° in the absence of organic compounds) (63). The genome of strain V6Fe1^T did not contain critical genes of the rTCA cycle but contained the complete set of TCA genes cycle, similar to those of *D. autotrophicus* and *G. thiophilus*. Strain V6Fe1^T could be another candidate for testing experimentally the reversed oxidative TCA cycle (ro TCA cycle), which is difficult to infer from genomic data. For example, *D. essentukiensis, C. nitroreducens,* and *D. desulfuricans* possess all the genes putatively involved in autotrophic carbon fixation in *D. autotrophicus* and strain V6Fe1^T. In contrast, those strains could not grow autotrophically with several electron acceptors (53)(54)(26).

Autotrophy is favored in cultures under high CO_2 concentrations, which drive the TCA cycle backward towards autotrophy, as was shown in *Hippea maritima* cultures (64). High CO_2 fluxes are usually measured at hydrothermal vents (65), especially in the Vulcano system (2). Autotrophy could then be an adaptive advantage for strain V6Fe1^T in this environment.

3.4 Sulfur and Iron respiratory genes

Strain V6Fe1^T can reduce elemental sulfur (S°) to sulfide, oxidizing various organic compounds (proteinaceous compounds, alcohols, and organic acids) or H_2. Sulfate or thiosulfate were not used as electron acceptors in any tested condition. Strain V6Fe1^T is unable to perform sulfide oxidation. Among the closest related strains, *D. essentukiensis, D. desulfuricans*, and *D. autotrophicus* also reduce S° into sulfide, *whereas C. nitroreducens does not, whereas this ability has not been tested for P. organivorans; the latter is unique*, within *Deferribacteraceae* as it reduces sulfate into sulfide.

V6Fe1^T may reduce elemental sulfur to sulfide *via* polysulfide reductases. The genome encodes two operons, including NrfD polysulfide reductases (DSN97_01825 and DSN97_11315), which are repressed when nitrate is supplied in the medium (see section 3.5). These operons have been found in *D. essentukiensis, D. autotrophicus,* and *D. desulfuricans*. *The C. nitroreducens* genome encompasses only one nrfD gene (CALNI_RS00910) but no duplication. These operons are represented in Figure 4.

In another operon, the gene coding the subunit B of a molybdopterin tetrathionate reductase (Ttr, DSN97_01830), an enzyme that reduces tetrathionate to thiosulfate, is associated with molybdopterin oxidoreductase subunits (DSN97_01830). Like in the *D. desulfuricans* genome, this operon is adjacent to another operon containing an outer membrane phosphate selective porin DSN97_01845 and a rhodanese domain-containing protein DSN97_01850. These two proteins may facilitate sulfur respiration in strain V6Fe1^{T,} acting as a polysulfide-specific porin in the outer membrane and a polysulfide-binding protein in the periplasmic space, respectively. Strain V6Fe1^T may also reduce sulfur thanks to a sulfide-dehydrogenase (DSN97_09915), also encoded in *D. essentukiensis* (LF845_RS10455), *C. nitroreducens* (CALNI_RS05465), *D. desulfuricans* (DEFDS_RS03360) and *D. autotrophicus* genomes.

Strain V6Fe1^T can reduce Fe(III) insoluble minerals but not soluble forms. In the *Deferribacterota* phylum, *D. essentukiensis* and *D. autotrophicus* are the only members capable of iron reduction whose genomes have been sequenced (53)(28). However, in the V6Fe1^T genome, as in the *D. autotrophicus* genome, homologs of the outer- or inner-membrane cytochromes MtrA, MtrC, OmcS, ImcH, and CbcL, MtrB, and OmbB, known to determine electron transfer in the model organism *S.*

oneidensis were not found. The Strain V6Fe1^T genome also does not contain genes encoding the proteins of flavin-based extracellular electron transfer in Gram-positive bacteria (66).

Nevertheless, strain V6Fe1^T genome possesses 14 genes coding multiheme c-type cytochromes whose role is difficult to assess. We found the gene coding for the quinone-converting enzyme CymA (DSN97_06310). Its specific cofactor, the menaquinone 7 MK-7 (58), was detected by biochemical methods (see 3.1), and a menaquinone biosynthesis methyltransferase gene (DSN97_04395) is also present in the genome. CymA is a monotopic membrane tetraheme c-type cytochrome belonging to the NapC/NirT family and central to anaerobic respiration in *Shewanella* sp. CymA could be key in shutting electrons to Fe(III) outside the cell *via* a small tetraheme cytochrome (STC) as a periplasmic shuttle (67). Our experiments on nitrate reduction revealed the involvement of CymA in fumarate degradation (3.5). Strain V6Fe1^T genome contains 17 genes encoding putative multiheme c-type cytochromes and 'e-pilin' genes (two genes encoding type IV pilins PilA DSN97_09015 and DSN97_02165), some of which are probably involved in Fe(III) reduction (67). A cytochrome c (DSN97_05235) closely related to the octaheme cytochrome of *D. autotrophicus* (FHQ18_RS08740) and the octaheme cytochrome of *D. essentukiensis* (LF845_RS02975) was found. This cytochrome was shown to be upregulated in the presence of ferrihydrite in a thermophilic Gram-positive bacterium (68) and proposed to be a key determinant of iron reduction in *Deferribacterales* as *D. desulfuricans* and *C. nitroreducens,* which are unable to perform iron reduction, lack it. The iron reduction could also be performed via a ferrireductase-like transmembrane domain-containing protein (DSN97_02000) adjacent to an energy-conserving hydrogenase (DSN97_02005). This protein has no homolog in other *Deferribacteraceae* genomes.

3.5 Nitrogen metabolism and metabolic pathways involved in nitrate reduction

Strain V6Fe1^T can use nitrate as an electron acceptor with numerous electron donors, heterotrophically and autotrophically. In the strain V6Fe1^T genome, we found the periplasmic nitrate reductase *napA* gene DSN97_05245 in an operon with genes coding a multiheme cytochrome c, adjacent to another operon containing the genes of chaperone protein *nap*D, *nap*G, and those coding a ferredoxin protein from the NapH/MauN family (Fig 5). This gene organization is in synteny with the same gene clusters found in the genomes of *D. essentukiensis, D. autotrophicus*, and *D. desulfuricans*. NapG and

NapH subunits are assumed to form an energy-conserving quinol dehydrogenase functioning as either a proton pump or in a Q cycle (69). The gene for the membrane-bound NarG nitrate reductase was not found in the genome. As NapA has a higher affinity for nitrate compared with that of the membrane-bound enzyme NarG (70), its occurrence in strain V6Fe1^T may represent an adaptation to the low nitrate concentrations typically found in vent fluids (13).

Strain V6Fe1^T genome does not contain assimilatory (*nirA*, *nirB*) or dissimilatory (*nirK*) nitrite reductase genes. Nevertheless, a formate-dependent nitrite reductase gene *nrfA* (DSN97_03745) and a *nrf*D gene (present in two adjacent reversed copies, DSN97_01825 and DSN97_11315) were identified, which could be involved in the respiratory ammonification (49). No nitric oxide reductase (*nos*Z) was found in the genome. The hem-containing (cd1-NIR, *nirS*) and the copper-containing (Cu-NIR, *nirK*) nitrite reductases genes catalyzing the reduction of nitrite (NO_2^-) to nitric oxide (NO) in most nitrate-reducing bacteria were absent in the V6Fe1^T genome. However, the presence of the gene *hcp* (hybrid cluster protein) DSN97_01195 possibly explains the reduction of nitric oxide to nitrous oxide or hydroxylamine to ammonium. A gene encoding a subunit of the anaerobic nitric oxide reductase flavorubredoxin NorV DSN97_07985 was found, which could also be involved in the reduction of nitric oxide to nitrous oxide. No enzyme linked to the oxidative part of the nitrogen cycle, nor any enzyme involved in dinitrogen fixation (except a NifU family protein, DSN97_10775) was identified in the genome.

Cultures with ^{15}N-labelled nitrate were performed to monitor the fate of nitrate during its reduction. The natural abundance of the N isotope is scarce (0.36%), so heavy values of $^{15}N\text{-}NH_4^+$ and $^{15}N\text{-}N_2$ detected in the culture medium are exclusively caused by the reduction of $^{15}NO_3$—by strain V6Fe1^T.

The standard isotopic ratio of atmospheric nitrogen is 0.366 ± 0.001 (71), close to the glycine standard we used. Heavier ratios showed enrichment of the heavy ^{15}N isotope, indicating the fate of ^{15}N-labelled nitrate (Table 2). Strong enrichment of both N_2 and NH_3 molecules was frequently observed, which proves the formation of isotopically heavy dinitrogen gas and ammonium from nitrate. Culture experiments in which nitrate was limiting (2 mM ^{15}N nitrate with 20 mM fumarate) showed enrichment in NH_4^+ ($^{15}N/^{14}N$ = 0.516), whereas not in N_2 ($^{15}N/^{14}N$ = 0.365). When fumarate and nitrate were

furnished at comparable concentrations (20 mM vs. 22 mM), ^{15}N enrichment was observed in both N_2 and NH_4^+ pools ($^{15}N/^{14}N$ = 0.492 and 1.36, respectively). Culture in autotrophic conditions showed less pronounced but significant enrichments, regardless of the NO_3^- concentrations. $^{15}N/^{14}N$ ratios in N_2 and NH_4^+ were between 0.377 and 0.372 and between 0.407 and 0.443 with 2 mM or 20 mM NO_3^-. Dinitrogen and ammonium isotopic ratios were linked to the concentration and nature of electron donors (fumarate or hydrogen) and acceptor (nitrate). These results showed that strain V6Fe1^T can use both pathways of nitrate reduction denitrification and DNRA. The relative importance of these pathways seems to depend on the electron donor's nature and concentration.

As these measures were taken as end-point results, on cultures having reached the stationary phase, new culture experiments were performed with fumarate 15 mM (condition FN) or dihydrogen (in H_2/CO_2, 80/20, v/v 1 bar) (condition HN) and nitrate 10 mM as the electron acceptor. Monitoring the concentrations in nitrite, nitrate, dihydrogen, carbon dioxide, nitrous oxide, fumarate, and succinate was used to specify the stoichiometry of the metabolic reactions and then identify the metabolic pathway involved in nitrate reduction (Fig. 6). For comparison, strain V6Fe1^T was cultivated in parallel in fermentative conditions (F) with fumarate 15 mM alone, used as the reference conditions in qRT-PCR analyses. Cells grew well in all conditions, entering the stationary phase at densities up to $1{,}8.10^9$ (F condition), $3{,}4.10^8$ cells. mL^{-1} (FN conditions) and $1{,}5.10^8$ cells. mL^{-1} (HN conditions). Differences between cell yield in fermentative and respiratory conditions may be due to the toxicity of nitrite accumulation in the medium (see section 3.1). Monitoring the concentrations of nitrite, nitrate, dihydrogen, carbon dioxide, nitrous oxide, fumarate, and succinate was used to specify the stoichiometry of the metabolic reactions and identify the metabolic pathway involved in nitrate reduction.

Cells were sampled in the middle exponential phase for RNA extractions and RT-qPCR experiments, as represented by the black arrows in Figure 6. This figure indicates the evolution of major metabolic reactants or products.

In the reference condition (F condition), succinate was produced while fumarate concentrations decreased. The stoichiometry of the reaction was about 2 moles of succinate formed per 3 moles of fumarate consumed. This stoichiometry agrees with previous results acquired on *Clostridium formicoaceticum* (72): 3 fumarate + 2 H2O → 2 succinate + 1 acetate + 2 CO2, corresponding to the

fumarate dismutation. For the fumarate plus nitrate (FN) condition, the theoretical denitrification reaction was 5 $C_4H_4O_4$ + 12 NO_3^- + 12 H^+ → 20 CO_2 + 6 N_2 + 16 H_2O. No ammonium production was observed (ammonium concentrations stayed between 1.4 and 1.7 mM during the FN experiments), and 2.07 to 2.35 moles of nitrate were consumed per mole of fumarate in the exponential phase, close to the theoretical stoichiometric ratio value of 2.4 according to the above equation. Nitrate was almost wholly consumed, dropping from 8 mM to 0.4 mM. As the ammonium concentration was stable in this condition, nitrate reduction was exclusively attributed to the respiratory denitrification of N_2. The discrepancy could be due to little fumarate dismutation as in the F condition, as suggested by the high expression of *cym*A (see below). In the hydrogen plus nitrate (HN) condition, no N_2O (denitrification intermediate) production was detected, as expected if denitrification occurred, while ammonium concentrations increased, indicative of the DNRA pathway. About one mole (0,89 to 0,99) of ammonium was produced per mole of nitrate consumed. Nitrate was also almost entirely consumed, as concentrations dropped from 7 mM to 0.3 mM. The DNRA reaction with dihydrogen can be written as 4 H_2 +NO_3^- +2 H^+ → NH_4^+ +3 H_2O. We assumed then that nitrate reduction with dihydrogen as the sole electron donor was exclusively performed by DNRA. In the exponential phase under our culture conditions (10 mM nitrate), only denitrification was observed with fumarate 15 mM as the carbon and energy source; only DNRA was observed with H_2/CO_2 as the carbon and energy source. These results can be interpreted according to thermodynamic considerations. Free Gibbs energies of DNRA and denitrification can be computed (12) and show that DNRA is more likely to happen when hydrogen reacts with nitrate ($\Delta G°$ = -662 kJ/mol NO_3^- for DNRA versus $\Delta G°$ = -580 kJ/mol NO_3^- for denitrification). Calculation of free energies of denitrification and DNRA with fumarate as the electron donor are less conclusive ($\Delta G°$ = -694 kJ/mol NO_3^- for DNRA versus $\Delta G°$ = -640 kJ/mol NO_3^- for denitrification).

On the one hand, DNRA yields more energy from one mole of nitrate, promoting this pathway when reducing power is in excess or effectively limiting nitrate. Thus, denitrification should be promoted in case of nitrate excess or limiting reducing power (73). Therefore, the differential expression of the genes involved in each pathway was measured to test this hypothesis. The results of the quantitative RT-PCR are summarized in Fig. 7.

Nitrate in the medium induced the nitrate reductase NapA. Gene expression was six times higher when nitrate was provided than in the control condition. So, the high-affinity periplasmic nitrate reductase should be the first step enzyme involved in nitrate reduction for both DNRA and denitrification.

In our experiments, *cym*A seemed repressed when nitrate was added, especially in the autotrophic condition. Transcription of *cym*A occurred only when fumarate was present in the medium. CymA transcription was observed in fumarate (F and FN conditions) during our experiments. Electrons should be transmitted to NapA *via* another electron shuttle, probably NapH. Usually, inner membrane-bound dehydrogenases pass electrons through the lipophilic menaquinone pool to CymA, transmitting them to periplasmic reductases. Results of *cym*A gene expression mean that MK-7 oxidation was coupled to the reduction of fumarate reductase (DSN97_05740, DSN97_05735), which reduces fumarate to succinate *via* CymA (74). Transcription of *cym*A during FN condition should account for the little discrepancy between the observed stoichiometry of nitrate consumption per mole of fumarate (2,07 to 2,35) compared to the theoretical ratio (2,4). CymA did not transfer electrons to the periplasmic nitrate reductase NapA or the nitrite reductase formate-dependent NrfA, as evidenced by qRT-PCR experiments.

Transcription of the *hcp* gene was high in HN conditions, 4.4 times the reference control, but 45 times higher than the control condition in FN conditions. Gene annotation indicated a possible role of the gene product in DNRA. The amino acid sequence of Hcp resembles that of a carbon monoxide dehydrogenase that can also reduce hydroxylamine (75). The HCP role has long been a matter of debate (76). The soluble Hcp protein in most bacteria reduces nitrate or nitrite anaerobically to ammonia but is absent from denitrifying *Alpha*- or *Beta-proteobacteria*. As demonstrated in (77) and (78), Hcp could assume a protective role against nitric oxide (NO) produced during nitrate reduction, which is higher in denitrification.

The *nrf*A relative expression was equivalent in F and FN conditions but twenty times higher in HN conditions. Thus, the pentaheme cytochrome c nitrite reductase (NrfA) may be the central enzyme catalyzing nitrite reduction to ammonium (79) by the DNRA pathway in strain V6Fe1^T. Electrons for nitrite reduction in V6Fe1^T strain could be furnished by a hydrogenase encoded in the genome

(DSN97_01270, DSN97_01760, DSN97_03610, DSN97_04825) via redox partners yet to be identified, perhaps a formate dehydrogenase encoded by DSN97_07560. NrfA-like cytochromes are also encoded in *D. essentukiensis* (LF845_RS03595), *D. desulfuricans* (DEFDS_RS06970), and *D. autotrophicus* (FHQ18_RS01740) in a syntenic organization, but not in *C. nitroreducens* genome. These cytochromes are probably involved in the complete nitrate reduction pathways. *D. desulfuricans,* on the contrary, only reduces nitrate to nitrite.

The *nor*V gene relative expression was equivalent in F and HN conditions and was repressed in FN conditions. It is annotated as a nitric oxide reductase and found in *D. essentukiensis, D. autotrophicus, C. nitroreducens,* and *D. desulfuricans* genomes, while these organisms do not use the denitrification pathway. Using the Blast algorithm, we found that this protein is classified as an FprA family A-type flavoprotein. Therefore, *norV* in strain V6Fe1^T may encode a ferredoxin NADP+ reductase involved in other redox reactions. The relative expression of *nrf*D was also surprising, as it was maximal in the F condition, quasi-null in the HN conditions, and intermediate in the FN condition. The periplasmic nitrite reductase (NrfABCD) was the first complex recognized to have a membrane subunit connected to the membrane through an integral membrane protein that interacts with quinones but does not have redox cofactors (80)(81). Sequence analyses indicated that NrfD homologs exist in many diverse enzyme families, such as polysulfide reductase (PsrABC) and other redox enzymes [80]. The reaction catalyzed by NrfD in strain V6Fe1^T could be sulfur reduction, as these enzymes are annotated as polysulfide reductase and are repressed when nitrate is added to the medium. Results of qRT-PCR experiments led to a conceptual model for nitrate reduction in strain V6Fe1^T represented in Figure 8, showing which genes were over- or under-expressed under our culture conditions, according to the nitrate reduction pathway considered.

In the *D. essentukiensis* and *D. autotrophicus* genomes, orthologous genes of strain V6Fe1^T, like *norV, napA,* and *hcp*, have been automatically annotated. *nrf*A has been retrieved by sequence alignment. These genes share the same environment in the genome. Slobodkin et al. (43) characterized the nitrate reduction pathways from genomic data and demonstrated that carbon monoxide was an electron donor for ammonification with culture experiments. These authors propose that *D. autotrophicus* performs DNRA with the involvement of *nap*A, *hao* (a hydroxylamine oxidoreductase

acting reversely), and *hcp.* Zavarzina et al. (53) propose that *D. essentukiensis* performs DNRA with an octaheme c hydroxylamine oxidoreductase of εHao group adjacently to the “Nap locus.” All these genes have also been found in the strain V6Fe1^T genome. Strain V6Fe1^T expressed *hcp* only when coping with denitrification, which cannot be performed in *D. essentukiensis* nor *D. autotrophicus*. Potential NrfA protein of strain V6Fe1^T shares 94.6% and 76.22 % sequence identities with a multiheme cytochrome c protein of *D. essentukiensis* (LF845_RS03595) and *D. autotrophicus* (FHQ18_RS01740), respectively, in the same genomic context. This multiheme cytochrome c domain-containing protein could be analogous to strain V6Fe1^T *nrf*A gene, also involved in DNRA. This raises doubts about the involvement of the gene with a *hao* (hydroxylamine oxidoreductase) annotation and *hcp* (hybrid cluster protein) in *D. essentukiensis* and *D. autotrophicus* DNRA hypothesized (43).

The switch between DNRA and denitrification in strain V6Fe1^T could be controlled by the balance between electron donor and electron acceptor availability, a large electron donor availability leading to the DNRA pathway. Adaptation is crucial in a changing environment such as the Vulcano hydrothermal system (82) and must result from the up- and downregulation of the same enzyme systems within the same organism. The genetic regulation of nitrate reduction pathways, e.g., denitrification or DNRA, has been investigated in different *Shewanella sp.* strains (83)(84)(85). This research emphasized the role of cyclic AMP receptor proteins (CRP). In *Shewanella* sp., transcription of DNRA-relevant genes such as nap-beta (*nap*DABGH), *nrf*A, and *cym*A genes were upregulated by CRP1, while *nir*K transcription was dependent on CRP2 (83). Strain V6Fe1^T exhibited different genes involved in nitrate reduction (absence of *nir*K, involvement of *cym*A in fumarate fermentation but not nitrate reduction, presence of only one periplasmic nitrite reductase). Nevertheless, strain V6Fe1^T's capacity to perform denitrification and DNRA could be related to two CRP proteins in its genome, DSN97_07130, and DSN97_07620.

Table 2. The isotopic ratio of dinitrogen and ammonium in the gas phase measured after one week of culture spiked with 2 mM $^{15}NO_3^-$.

Sample	($^{15}N/^{14}N$) of dinitrogen in the gas phase %	($^{15}N/^{14}N$) of ammonium in the liquid phase %
Glycocolle (reference)	0,367	0,367
Fumarate + 2 mM $^{15}NO_3^-$	0,365	0,516
Fumarate + 2 mM $^{15}NO_3^-$ + 20 mM NO_3^-	0,492	1,360
H_2/CO_2 80:20 1 bar + 2 mM $^{15}NO_3^-$	0,377	0,407
H_2/CO_2 80:20 1 bar + 2 mM $^{15}NO_3^-$ + 20 mM NO_3^-	0,372	0,443

5. Conclusion

Genomic and physiologic analyses of strain V6Fe1^T revealed several remarkable metabolic traits of this bacterium within the *Deferribacterota* phylum. Genomic data suggested that CO_2 assimilation occurs *via* an atypical, recently discovered, reversible oxidative TCA cycle (62)(64). Nitrate was reduced by both DNRA and denitrification pathways, thus being reduced to ammonium or dinitrogen depending on the reducing power/nitrate concentration balance. This balance could be regulated by the cyclic AMP binding proteins Crp1 and its Crp2 paralogue (83). The nitrate reduction system encompasses a soluble nitrate reductase NapA. NapA utilization should be related to the low nitrate concentrations in shallow hydrothermal systems. The conversion of nitrite to ammonium proceeded *via* the action of the formate-dependent nitrite reductase NrfA. The biochemical system of denitrification and anaerobic respirations in strain V6Fe1^T deserves further investigation.

Shallow hydrothermal systems have been recently the subject of numerous investigations (86)(87)(88) as they constitute easy access to extreme environments and may be actual analogs of places where life appeared (89). Indeed, due to the mixing of hot, reduced, and sometimes metal/sulfur-rich fluids with oxygenated seawater, these environments are characterized by high temperature and fluid chemistry gradients. Hydrothermal systems show rapid variation in the oxidation state of macro elements (carbon, sulfur, nitrogen, iron) in their surroundings (90), which offer various ecological niches. Due to its original metabolic capabilities and taxonomic affiliation, strain V6Fe1^T could be an attractive model for studying the adaptation of bacteria in changing environments like shallow subsurface hydrothermal fields.

Proposition for a new species

Strain V6Fe1^T and *Deferrivibrio essentukiensis* share many genomic traits and are closely related according to the 16S rRNA sequence (99.5 % identity). Given that strain V6Fe1^T can ferment proteinaceous substrates and organic acids, can grow lithoautotrophically with H_2/CO_2 as an energy and carbon source, and performs both denitrification and DNRA at the difference of its closest relatives, *Deferrivibrio essentukiensis* and *Petrothermobacter organivorans*, and based on their significant differences on growth optima (temperature, salinity and pH), doubling time and genomic ANI value, we propose strain V6Fe1^T (= DSM 27501^T = JCM 39088^T) to be the type strain of a novel species named *Deferrivibrio metallireducens*.

Description of *Deferrivibrio metallireducens* sp. nov. (me.tal'li.re.du'cens. L. n. metallum metal; L. part. adj. reducens leading back, bringing back, and, in chemistry, converting to a different oxidation state; N.L. part. adj. metallireducens reducing metal)

Small rods that are straight- to slightly vibrio-shaped. Cells are 4-8 µm long and 0.2-0.8 µm wide, with the Gram-negative type of cell wall. Non-motile. Anaerobic. Moderately thermophilic, growing between

Capital words used

45 and 65 °C, with optimum growth at 55-60 °C. Neutrophilic, growing between pH 5.1 and 7.7, with optimum growth at pH 6.3–6.5. Grows at NaCl concentrations ranging from 0 to 80 g/L, with optimum growth at 25 g/L NaCl. Capable of chemolithoautotrophic growth with hydrogen as an electron donor, manganese, insoluble ferric iron, elemental sulfur, or nitrate as electron acceptors, and CO_2 as a carbon source. Anaerobic oxidation of acetate, pyruvate, succinate, and proteinaceous substrates using sulfur, nitrate, Mn(IV), or insoluble Fe(III) as electron acceptors. Capable of fermentation yeast extract, peptone, and organic acids. Sensitive to ampicillin, bacitracin, chloramphenicol, cycloheximide, gentamicin, kanamycin, and tetracycline but resistant to vancomycin and streptomycin. Isolated on the seashore from sediments affected by hydrothermal circulations of the Vulcano hydrothermal system. The type strain is V6Fe1^T (= DSM 27501^T = JCM 39088^T). The G+C content of its DNA is 34.8 mol %. Isolated from the Vulcano (38°24'45'' N, 14° 57' 38'' E) hydrothermal system.

Acknowledgments:

The project X-TREMOPHILES (coordinator G. Erauso, 2017-2019) was founded by the program X-Life of the Interdisciplinarity Mission (MITI) of the CNRS (France). We thank Isabelle Canihac for strain isolation, Amandine Ruiz and Manon Joseph-Bartoli for help in culture experiments, Sophie Guasco for assistance in qRT-PCR experiments, and Patricia Bonin and Patrick Raimbault for assistance in isotopic analyses. We thank Giorgio Capasso and the INGV Institute for their help in sampling and geochemical analyses. The LABGeM (CEA/Genoscope & CNRS UMR8030), the France Génomique, and the French Bioinformatics Institute national infrastructures (funded as part of Investissement d'Avenir program managed by Agence Nationale pour la Recherche, contracts ANR-10-INBS-09 and ANR-11-INBS-0013) are acknowledged for support within the MicroScope annotation platform.

Bibliography

1. Stetter K.O., König H., and Stackebrandt E., *Pyrodictium* gen. nov., a New Genus of Submarine Disc-Shaped Sulphur Reducing Archaebacteria Growing Optimally at 105°C. Systematic and Applied Microbioly, 1983. 4(4): p. 535-51

2. Capasso G., Favara R., and Inguaggiato S., Chemical Features and Isotopic Composition of Gaseous Manifestations on Vulcano Island, Aeolian Islands, Italy: An Interpretative Model of Fluid Circulation. Geochimica et Cosmochimica Acta**,** 1997. 61(16): p. 3425–3440

3. Rusch A., Walpersdorf E., deBeer D., Gurrieri S., and Amend J.P., Microbial Communities near the Oxic/Anoxic Interface in the Hydrothermal System of Vulcano Island, Italy. Chemical Geology, 2005. 224(1-4): p. 169–182.

4. Skoog A., Vlahos P., Rogers K.L., and Amend J.P., Concentrations, Distributions, and Energy Yields of Dissolved Neutral Aldoses in a Shallow Hydrothermal Vent System of Vulcano, Italy. Organic Geochemistry, 2007. 38: p. 1416–1430.

5. Huber R., and Stetter K.O., *Aquifex*. In: Whitman W.B., Rainey F., Kämpfer P., Trujillo M., Chun J., DeVos P., et al., eds. Bergey's Manual of Systematics of Archaea and Bacteria. 1st ed. Wiley; 2015. p. 1- 5

6. Huber R., Langworthy T.A., König H., Thomm M., Woese C.R., Sleytr U.B., et al., Thermotoga maritima sp. nov. represents a new genus of unique extremely thermophilic eubacteria growing up to 90°C. Arch Microbiol., 1986. 144(4): p. 324- 33.

7. Stetter K.O., Archaeoglobus fulgidus gen. nov., sp. nov.: a New Taxon of Extremely Thermophilic Archaebacteria. Systematic and Applied Microbiology, 1988. 10(2): p. 172- 3.

8. Hafenbradl D., Keller M., Dirmeier R., Rachel R., Roßnagel P., Burggraf S., et al., Ferroglobus placidus gen. nov., sp. nov., a novel hyperthermophilic archaeum that oxidizes Fe 2+ at neutral pH under anoxic conditions. Archives of Microbiology, 1996. 166(5): p. 308- 14.

9. Amend J.P., Meyer-Dombard D.R., Sheth S.N., Zolotova N., and Amend A.C., Palaeococcus helgesonii sp. nov., a facultatively anaerobic, hyperthermophilic archaeon from a geothermal well on Vulcano Island, Italy. Arch Microbiol., 2003. 179(6): p. 394- 401.

10. Zillig W., Holz I., Janekovic D., Schäfer W., and Reiter W.D., The Archaebacterium Thermococcus celer Represents, a Novel Genus within the Thermophilic Branch of the Archaebacteria. Systematic and Applied Microbiology, 1983. 4(1): p. 88- 94.

Scientific word italics

11. Stetter K.O., *Staphylothermus*. In: Whitman W.B., Rainey F., Kämpfer P., Trujillo M., Chun J., DeVos P., et al., eds. Bergey's Manual of Systematics of Archaea and Bacteria. 1st ed. Wiley; 2015: p. 1- 3.

12. Amend J.P., Rogers K.L., Shock E.L., Gurrieri S., and Inguaggiatoa S., Energetics of chemolithoautotrophy in the hydrothermal system of Vulcano Island, southern Italy. Geobiology, 2003. 1(1): p. 37-58.

13. Schutte C.A., Teske A., MacGregor B.J., Salman-Carvalho V., Lavik G., Hach P., et al., Filamentous Giant Beggiatoaceae from the Guaymas Basin Are Capable of both Denitrification and Dissimilatory Nitrate Reduction to Ammonium. Appl Environ Microbiol., 2018. 84(15).

14. Vetriani C., Voordeckers J.W., Crespo-Medina M., O'Brien C.E., Giovannelli D., and Lutz R.A., Deep-sea hydrothermal vent Epsilonproteobacteria encode a conserved and widespread nitrate reduction pathway (Nap). ISME J., 2014. 8(7): p. 1510- 21.

15. Kuypers M.M.M., Marchant H.K., and Kartal B., The Microbial Nitrogen-Cycling Network. Nat Rev Microbiol., 2018. 16(5): p. 263–276

16. Machida T., Nakazawa T., Fujii Y., Aoki S., and Watanabe O., Increase in the atmospheric nitrous oxide concentration during the last 250 years. Geophysical Research Letters, 1995. 22(21): p. 2921-2924.

Maintain reference formate

17. Slobodkin A.I., Reysenbach A.L., Slobodkina G.B., Baslerov R.V., Kostrikina N.A., Wagner I.D. et al., Thermosulfurimonas dismutans gen. nov., sp. nov., an extremely thermophilic sulfur-disproportionating bacterium from a deep-sea hydrothermal vent. Int J Syst Evol Microbiol., 2012. 62(11): p. 2565-2571.

18. Slobodkin A.I., Reysenbach A.L., Slobodkina G.B., Kolganova T.V., Kostrikina N.A. and Bonch-Osmolovskaya E.A., Dissulfuribacter thermophilus gen. nov., sp. nov., a thermophilic, autotrophic, sulfur-disproportionating, deeply branching deltaproteobacterium from a deep-sea hydrothermal vent. International Journal of Systematic and Evolutionary Microbiology, 2013. 63(6): p. 1967- 71.

All scientific name should be Italic.

19. McAllister S.M., Moore R.M., Gartman A., Luther G.W., Emerson D., and Chan C.S., The Fe(II)-oxidizing *Zetaproteobacteria*: historical, ecological and genomic perspectives. FEMS Microbiology Ecology. 2019. 95(4).

20. Miroshnichenko M.L., Kostrikina N.A., Chernyh N.A., Pimenov N.V., Tourova T.P., Antipov A.N., et al., Caldithrix abyssi gen. nov., sp. nov., a nitrate-reducing, thermophilic, anaerobic bacterium isolated from a Mid-Atlantic Ridge hydrothermal vent, represents a novel bacterial lineage. International Journal of Systematic and Evolutionary Microbiology, 2003. 53(1): p. 323- 9.

21. Pérez-Rodríguez I., Ricci J., Voordeckers J.W., Starovoytov V., and Vetriani C., Nautilia nitratireducens sp. nov., a thermophilic, anaerobic, chemosynthetic, nitrate-ammonifying bacterium isolated from a deep-sea hydrothermal vent. International Journal of Systematic and Evolutionary Microbiology, 2010. 60(5): p. 1182- 6.

22. Slobodkina G.B., Reysenbach A.L., Kolganova T.V., Novikov A.A., Bonch-Osmolovskaya E.A. and Slobodkin A.I., *Thermosulfuriphilus ammonigenes* gen. nov., sp. nov., a thermophilic,

chemolithoautotrophic bacterium capable of respiratory ammonification of nitrate with elemental sulfur. Int J Syst Evol Microbiol., 2017. 67(9): p. 3474-3479.

23. L'Haridon S., Miroshnichenko M.L., Kostrikina N.A., Tindall B.J., Spring S., Schumann P., et al., Vulcanibacillus modesticaldus gen. nov., sp. nov., a strictly anaerobic, nitrate-reducing bacterium from deep-sea hydrothermal vents. International Journal of Systematic and Evolutionary Microbiology, 2006. 56(5): p. 1047- 53.

24. Takai K., Suzuki M., Nakagawa S., Miyazaki M., Suzuki Y., Inagaki F., et al., Sulfurimonas paralvinellae sp. nov., a novel mesophilic, hydrogen- and sulfur-oxidizing chemolithoautotroph within the Epsilonproteobacteria isolated from a deep-sea hydrothermal vent polychaete nest, reclassification of Thiomicrospira denitrificans as Sulfurimonas denitrificans comb. nov. and emended description of the genus Sulfurimonas. International Journal of Systematic and Evolutionary Microbiology, 2006. 56(8): p. 1725- 33.

25. Nakagawa S., Takai K., Inagaki F., Horikoshi K., and Sako Y., Nitratiruptor tergarcus gen. nov., sp. nov. and Nitratifractor salsuginis gen. nov., sp. nov., nitrate-reducing chemolithoautotrophs of the ε-Proteobacteria isolated from a deep-sea hydrothermal system in the Mid-Okinawa Trough. International Journal of Systematic and Evolutionary Microbiology, 2005. 55(2): p. 925- 33.

26. Takai K., Kobayashi H., Nealson K.H. and Horikoshi, K., *Deferribacter desulfuricans* sp. nov., a novel sulfur-, nitrate- and arsenate-reducing thermophile isolated from a deep-sea hydrothermal vent. Int J Syst Evol Microbiol., 2003. 53(3): p. 839-846.

27. Tamazawa S., Mayumi D., Mochimaru H., Sakata S., Maeda H., Wakayama T., et al., *Petrothermobacter organivorans* gen. nov., sp. nov., a thermophilic, strictly anaerobic bacterium of the phylum Deferribacteres isolated from a deep subsurface oil reservoir. Int J Syst Evol Microbiol., 2017. 67(10): p. 3982-3986.

28. Slobodkina G.B., Kolganova T.V., Chernyh N.A., Querellou J., Bonch-Osmolovskaya E.A., and Slobodkin A.I., *Deferribacter autotrophicus* sp. nov., an iron(III)-reducing bacterium from a deep-sea hydrothermal vent. Int J Syst Evol Microbiol., 2009. 59(6): p. 1508-12.

29. Miroshnichenko M.L., Slobodkin A.I., Kostrikina N.A., L'Haridon S., Nercessian O., Spring, S., et al., *Deferribacter abyssi* sp. nov., an anaerobic thermophile from deep-sea hydrothermal vents of the Mid-Atlantic Ridge. Int J Syst Evol Microbiol., 2003. 53(5): p. 1637-1641.

30. Balch W.E., Fox G.E., Magrum L.J., Woese C.R., and Wolfe, R.S., Methanogens: Reevaluation of a Unique Biological Group. Microbiol. Rev., 1979. 43(2): p. 260-96.

31. Hungate, R.E., Chapter IV A Roll Tube Method for Cultivation of Strict Anaerobes. In Methods in Microbiology, 1969. 3: 117-132.

32. Thabet O.B.D., Fardeau M.L., Joulian C., Thomas P., Hamdi M., Garcia J.L., et al., Clostridium tunisiense sp. nov., a new proteolytic, sulfur-reducing bacterium isolated from an olive mill wastewater contaminated by phosphogypse. Anaerobe, 2004. 10(3): p. 185- 90.

33. Altschul S.F., Gish W., Miller W., Myers E.W., and Lipman D.J., Basic local alignment search tool. Journal of Molecular Biology, 1990. 215(3): p. 403- 10.

34. Edgar R.C., MUSCLE: multiple sequence alignment with high accuracy and high throughput. Nucleic Acids Research, 2004. 32(5): p. 1792- 7.

35. Kumar S., Stecher G., and Tamura K., MEGA7: Molecular Evolutionary Genetics Analysis Version 7.0 for Bigger Datasets. Molecular Biology and Evolution, 2016. 33(7): p. 1870- 4.

36. Erauso G., Reysenbach A.L., Godfroy A., Meunier J.R., Crump B., Partensky F., et al., *Pyrococcus abyssi* sp. nov., a new hyperthermophilic archaeon isolated from a deep-sea hydrothermal vent. Arch Microbiol., 1993. 160: p. 338–349.

37. Vallenet D., Calteau A., Cruveiller S., Gachet M., Lajus A., Josso A., et al., MicroScope in 2017: an expanding and evolving integrated resource for community expertise of microbial genomes. Nucleic Acids Res., 2017. 45(D1): p. 517- 28.

38. Hitch T.C.A., Riedel T., Oren A., Overmann J., Lawley T.D., and Clavel T., Automated analysis of genomic sequences facilitates high-throughput and comprehensive description of bacteria. ISME COMMUN., 2021. 1(1): p. 16.

39. Xu L., Dong Z., Fang L., Luo Y., Wei Z., Guo H., et al., OrthoVenn2: a web server for whole-genome comparison and annotation of orthologous clusters across multiple species. Nucleic Acids Research, 2019. 47(W1): p. 52- 8.

40. Katoh, K., and Standley, D.M., MAFFT multiple sequence alignment software version 7: improvements in performance and usability. Mol Biol Evol., 2013. 30: p. 772-80.

41. Minh B.Q., Schmidt H.A., Chernomor O., Schrempf D., Woodhams M.D., von Haeseler, A., et al., IQ-TREE 2: New Models and Efficient Methods for Phylogenetic Inference in the Genomic Era. Mol Biol Evol., 2020. 37(5): p. 1530-1534.

42. Chaumeil P.A., Mussig A.J., Hugenholtz P., and Parks D.H., GTDB-Tk: a toolkit to classify genomes with the Genome Taxonomy Database. Hancock J, ed. Bioinformatics, 2020;36(6): p. 1925- 7.

43. Slobodkin A., Slobodkina G., Allioux M., Alain K., Jebbar M., Shadrin V., et al., Genomic Insights into the Carbon and Energy Metabolism of a Thermophilic Deep-Sea Bacterium Deferribacter autotrophicus Revealed New Metabolic Traits in the Phylum Deferribacteres. Genes, 2019. 10(11): p. 849.

44. Fukushi M., Mino S., Tanaka H., Nakagawa S., Takai K., and Sawabe T., Biogeochemical Implications of N_2O-Reducing Thermophilic *Campylobacteria* in Deep-Sea Vent Fields, and the Description of *Nitratiruptor labii sp. nov*. iScience, 2020. 23: p. 101462.

45. Fardeau M.L., Ollivier B., Patel B.K.C., Magot M.T; Thomas, P., Rimbault, A., et al., *Thermotoga hypogea* sp. nov., a Xylanolytic, Thermophilic Bacterium from an Oil-Producing Well. Int J Syst Evol Microbiol., 1997. 47(4): p. 1013-1019.

46. Kuykendall L.D., Roy M.A., O'Neil J.J., and Devine T.E., Fatty Acids, Antibiotic Resistance, and Deoxyribonucleic Acid Homology Groups of *Bradyrhizobiurn Japonicum.* Int J Syst Evol Microbiol., 1988. 4(38): p. 358-361.

47. Harwood J.E., and Huyser D.J., Automated Analysis of Ammonia in Water. Water Research, 1970. 4(10): p. 695–704.

48. Slawyk G., and Raimbault P., Simple procedure for simultaneous recovery of dissolved inorganic and organic nitrogen in ^{15}N-tracer experiments and improving the isotopic mass balance. Mar Ecol Prog Ser., 1995. 124(1-3): p. 289-299.

49. Gao H., Yang Z.K., Barua S., Reed S.B., Romine M.F., Nealson K.H., et al., Reduction of nitrate in Shewanella oneidensis depends on atypical NAP and NRF systems with NapB as a preferred electron transport protein from CymA to NapA. ISME J., 2009. 3(8): p. 966- 76.

50. Muyzer G., de Waal E.C., and Uitterlinden A.G., Profiling of complex microbial populations by denaturing gradient gel electrophoresis analysis of polymerase chain reaction-amplified genes coding for 16S rRNA. Appl Environ Microbiol., 1993. 59(3): p. 695- 700.

51. Takai K., and Horikoshi K., Rapid Detection and Quantification of Members of the Archaeal Community by Quantitative PCR Using Fluorogenic Probes. Appl Environ Microbiol., 2000. 66(11): p. 5066- 72.

52. Beccaluva L., Gabbianelli G., Lucchini F., Rossi P.L., and Savelli C. Petrology and K/Ar ages of volcanics dredged from the Eolian seamounts: implications for geodynamic evolution of the southern Tyrrhenian basin. Earth and Planetary Science Letters, 1985. 74(2- 3): p. 187- 208.

53. Zavarzina D.G., Prokofeva M.I., Pikhtereva V.A., Klyukina A.A., Maslov A.A., Merkel A.Yu., et al., Deferrivibrio essentukiensis sp. nov., gen. nov., a Representative of Deferrivibrionaceae fam. nov., Isolated from the Subsurface Aquifer of Caucasian Mineral Drinking Waters Microbiology, 2022. 91: p. 143–159

54. Iino T., Nakagawa T., Mori K., Harayama S., and Suzuki K., Calditerrivibrio nitroreducens gen. nov., sp. nov., a thermophilic, nitrate-reducing bacterium isolated from a terrestrial hot spring in Japan. Int J Syst Evol Microbiol., 2008. 58(7): p. 1675- 9.

55. Greene A.C., Patel B.K.C., and Sheehy A.J., *Deferribacter thermophilus* Gen. Nov., Sp. Nov., a Novel Thermophilic Manganese- and Iron-Reducing Bacterium Isolated from a Petroleum Reservoir. Int J Syst Evol Microbiol., 1997. 47(2): p. 505–509.

56. de Carvalho C.C.C.R., Marques M.P.C., Hachicho N., and Heipieper H.J., Rapid adaptation of Rhodococcus erythropolis cells to salt stress by synthesizing polyunsaturated fatty acids. Appl Microbiol Biotechnol., 2014. 98(12): p. 5599-606.

57. Spring S., Brinkmann N., Murrja M., et al., High diversity of culturable prokaryotes in a lithifying hypersaline microbial mat. Geomicrobiol J. 2015. 32: p. 332–346.

58. McMillan D.G.G., Marritt S.J., Butt J.N., and Jeuken L.J.C. Menaquinone-7 Is Specific Cofactor in Tetraheme Quinol Dehydrogenase CymA. Journal of Biological Chemistry 2012;287(17): p. 14215- 25.

59. Chun J., Oren A., Ventosa A., Christensen H., Arahal D.R., da Costa M.S., et al. Proposed minimal standards for the use of genome data for the taxonomy of prokaryotes. International Journal of Systematic and Evolutionary Microbiology, 2018. 68(1): p. 461- 6.

60. Qin Q.L., Xie B.B., Zhang X.Y., Chen X.L., Zhou B.C., Zhou J., et al. A Proposed Genus Boundary for the Prokaryotes Based on Genomic Insights. J Bacteriol., 2014. 196(12): p. 2210- 5.

61. Drula E., Garron M.L., Dogan S., Lombard V., Henrissat B., and Terrapon N., The carbohydrate-active enzyme database: functions and literature. Nucleic Acids Research, 2022. 50(D1): D571- 7.

62. Mall A., Sobotta J., Huber C., Tschirner C., Kowarschik S., Bačnik K., et al., Reversibility of citrate synthase allows autotrophic growth of a thermophilic bacterium. Science, 2018. 359(6375): p. 563- 7

63. Janssen P.H., Geovibrio thiophilus sp. nov., a novel sulfur-reducing bacterium belonging to the phylum Deferribacteres. Int J Syst Evol Microbiol., 2002. 52(4): p. 1341- 7.

64. Steffens L., Pettinato E., Steiner T.M., Mall, A., König S., Eisenreich W., et al., High CO_2 Levels Drive the TCA Cycle Backwards towards Autotrophy. Nature, 2021. 592(7856): p. 784-788.

65. Dahle H., Le Moine Bauer S., Baumberger T., Stokke R., Pedersen, R.B., Thorseth, I.H., et al., Energy Landscapes in Hydrothermal Chimneys Shape Distributions of Primary Producers. Front. Microbiol. 2018. 16(9): 1570.

66. Light S.H., Su L., Rivera-Lugo R., Cornejo J.A., Louie A., Iavarone A.T., et al., A flavin-based extracellular electron transfer mechanism in diverse Gram-positive bacteria. Nature, 2018. 562(7725): p. 140- 4.

67. Qian Y., Paquete C.M., Louro R.O., Ross D.E., LaBelle E., Bond D.R, et al., Mapping the Iron Binding Site(s) on the Small Tetraheme Cytochrome of *Shewanella oneidensis* MR-1. Biochemistry, 2011. 50(28): p. 6217- 24.

68. Gavrilov S.N., Zavarzina D.G., Elizarov I.M., Tikhonova T.V., Dergousova N.I., Popov O., et al., Novel extracellular electron transfer channels in a Gram-positive thermophilic bacterium. Front. Microbiol., 2021. 11:597818.

69. Brondijk T.H.C., Fiegen D., Richardson D.J., and Cole J.A., Roles of NapF, NapG and NapH, Subunits of the Escherichia Coli Periplasmic Nitrate Reductase, in Ubiquinol Oxidation: Roles of NapF, G and H in Ubiquinol Oxidation in E. Coli. Molecular Microbiology, 2002. 44(1): p. 245-55.

70. Potter L.C., Millington P., Griffiths L., Thomas G.H., and Cole, J.A., Competition between Escherichia Coli Strains Expressing Either a Periplasmic or a Membrane-Bound Nitrate Reductase: Does Nap Confer a Selective Advantage during Nitrate-Limited Growth ? Biochem J., 1999. 344(1): p. 77-84.

71. Brand W.A., Coplen T.B., Vogl J., Rosner M., and Prohaska T., Assessment of international reference materials for isotope-ratio analysis (IUPAC Technical Report). Pure and Applied Chemistry, 2014. 86(3): p. 425- 67.

72. Dorn M., Andreesen J.S., and Gottschalk G., Fermentation of Fumarate and L-Malate by *Clostridium formicoaceticum.* J. Bact., 1978. 133(1): p. 26-32.

73. Strohm T.O., Griffin B., Zumft W.G., and Schink B., Growth Yields in Bacterial Denitrification and Nitrate Ammonification. Appl Environ Microbiol., 2007. 73(5): p. 1420- 4.

74. Schwalb C., Chapman S.K., and Reid G.A., The membrane-bound tetrahaem c-type cytochrome CymA interacts directly with the soluble fumarate reductase in *Shewanella*. Biochemical Society Transactions, 2002. 30(4): p. 658- 62.

75. Wolfe M.T., Heo J., Garavelli J.S., and Ludden P.W. Hydroxylamine Reductase Activity of the Hybrid Cluster Protein from *Escherichia coli*. J Bacteriol., 2002. 184(21): p. 5898- 902.

76. Cole J.A., Anaerobic Bacterial Response to Nitrosative Stress. In: Advances in Microbial Physiology, Elsevier. 2018. p. 193- 237.

77. Boutrin M.C., Wang C., Aruni W., Li X., and Fletcher H.M., Nitric Oxide Stress Resistance in Porphyromonas gingivalis Is Mediated by a Putative Hydroxylamine Reductase. J Bacteriol., 2012. 194(6): p. 1582- 92.

78. Meng Q., Yin J., Jin M., and Gao H. Distinct Nitrite and Nitric Oxide Physiologies in Escherichia coli and Shewanella oneidensis. Appl Environ Microbiol., 2018. 84(12): e00559-18.

79. Einsle O., Structure and Function of Formate-Dependent Cytochrome c Nitrite Reductase, NrfA. In: Methods in Enzymology, Elsevier. 2011. p. 399- 422.

80. Simon J., Enzymology and bioenergetics of respiratory nitrite ammonification. FEMS Microbiol Rev., 2002. 26(3): p. 285-309.

81. Sousa F.M., Pereira J.G., Marreiros B.C., and Pereira M.M., Taxonomic Distribution, Structure/Function Relationship and Metabolic Context of the Two Families of Sulfide Dehydrogenases: SQR and FCSD. Biochimica et Biophysica Acta (BBA) – Bioenergetics 2018 1859(9): p. 742–753.

82. Rogers K.L., Amend J.P., and Gurrieri S., Temporal Changes in Fluid Chemistry and Energy Profiles in the Vulcano Island Hydrothermal System. Astrobiology, 2007. 7(6): p. 905- 32.

83. Liu S., Dai J., Wei H., Li S., Wang P., Zhu T., et al., Dissimilatory Nitrate Reduction to Ammonium (DNRA) and Denitrification Pathways Are Leveraged by Cyclic AMP Receptor Protein (CRP) Paralogues Based on Electron Donor/Acceptor Limitation in Shewanella loihica PV-4. Appl Environ Microbiol., 2021. 87(2): e01964-20.

84. Chen Y., Wang F., Xu J., Mehmood M.A., and Xiao X., Physiological and evolutionary studies of NAP systems in Shewanella piezotolerans WP3. ISME J., 2011;5(5): p. 843- 55.

85. Dong Y., Wang J., Fu H., Zhou G., Shi M., and Gao H. A Crp-dependent two-component system regulates nitrate and nitrite respiration in Shewanella oneidensis. PLoS One, 2012. 7(12): e51643.

Proper alignment required

86. Pisapia C., Gérard E., Gérard M., Lecourt L., Lang S.Q., Pelletier B., et al. Mineralizing Filamentous Bacteria from the Prony Bay Hydrothermal Field Give New Insights into the Functioning of Serpentinization-Based Subseafloor Ecosystems. Front Microbiol., 8 :57.

87. Pop Ristova P., Pichler T., Friedrich M.W., and Bühring S.I., Bacterial Diversity and Biogeochemistry of Two Marine Shallow-Water Hydrothermal Systems off Dominica (Lesser Antilles). Front. Microbiol., 2017. 8: 2400.

88. Belilla J., Moreira D., Jardillier L., Reboul G., Benzerara, K., López-García J.M., et al., Hyperdiverse Archaea near Life Limits at the Polyextreme Geothermal Dallol Area. Nat Ecol Evol., 2019. 3(11): p. 1552-1561.

89. Westall F., Campbell K.A., Bréhéret J.G., Foucher F., Gautret P., Hubert A., et al., Archean (3.33 Ga) microbe-sediment systems were diverse and flourished in a hydrothermal context. Geology, 2015. 43 (7): 615–618.

90. Barge L.M., Flores E., Baum M.M., VanderVelde D.G., and Russell M.J., Redox and pH Gradients Drive Amino Acid Synthesis in Iron Oxyhydroxide Mineral Systems. Proc. Natl. Acad. Sci., 2019. 116(11): p. 4828-4833.

Figure

Click here to access/download;Figure;Figures_V6Fe1_24_juillet2024_PlosO

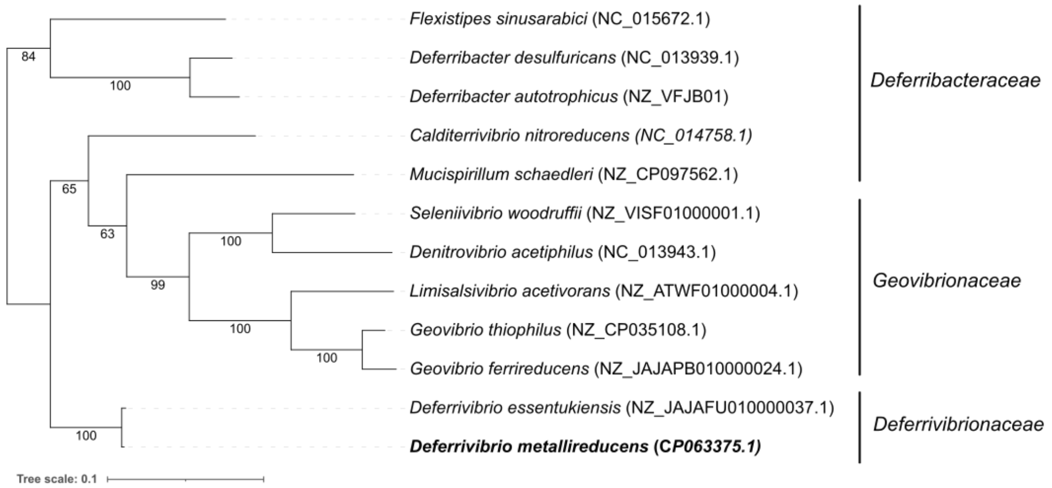

Figure 1. Phylogenetic tree based on 16S rRNA gene sequences showing the position of *Deferrivibrio metallireducens* V6Fe1^T in the family *Deferrivibrionaceae,* related to the families *Deferribacteraceae* and *Geovibrionaceae*. The outgroup (not represented here) was composed of the following type species: *Thermanaeromonas toyohensis* ToBE*, Moorella thermoacetica* DSM 2955*, Moorella humiferrea* OCP, *Moorella glycerini* DSM 11254, *Desulfonatronum thiosulfatophilum* ASO4-2, *Desulfonatronum thioautotrophicum* ASO4-1, *Desulfonatronum thiodismutans* MLF1, *Desulfonatronum lacustre* DSM 10312, *Desulfovibrio ferrophilus* IS5, *Desulfovibrio subterraneus* ND17, *Desulfovibrio mangrove* FT415, *Desulfovibrio desulfuricans* L4, *Desulfovibrio fairfieldensis* CCUG 45958, *Candidatus Desulfovibrio trichonymphae* Rs-N31. 16S rRNA gene sequences were extracted from genome assemblies using barrnap 0.9, and the multiple alignments of the sequences were performed with mafft 7.515 (40). The maximum-likelihood phylogenetic tree was generated using IQ-TREE 2.3.4 (41) with GTR+F+G4 model. Bootstraps are depicted at nodes, estimated by IQ-TREE -B 1000 option. The scale bar indicates the number of substitutions per site.

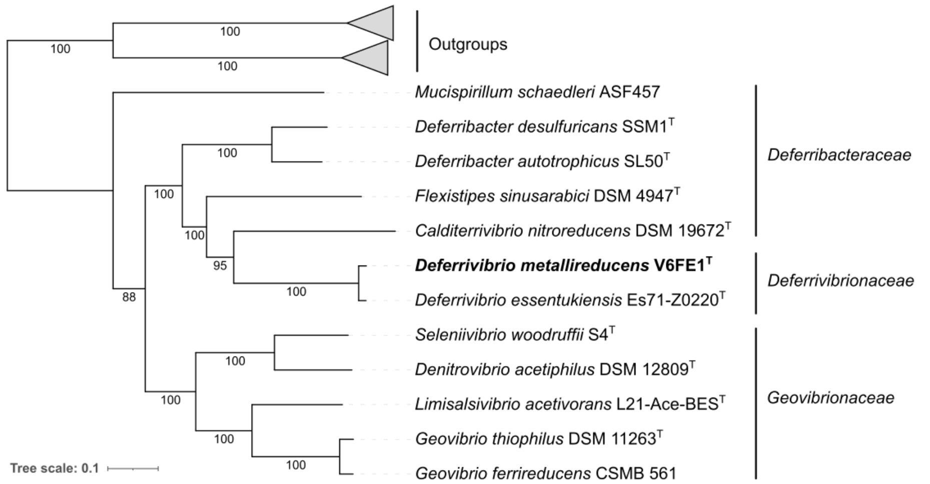

Figure 2. Phylogenetic tree based on 120 bacterial marker genes by GTDB-Tk showing the position of *Deferrivibrio metallireducens* V6FE1^T in the family *Deferrivibrionaceae,* related to the families *Deferribacteraceae* and *Geovibrionaceae*. The outgroup (not represented here) was composed of the following type species: *Thermanaeromonas toyohensis* ToBE*, Moorella thermoacetica* DSM 2955*, Moorella humiferrea* OCP, *Moorella glycerini* DSM 11254, *Desulfonatronum thiosulfatophilum* ASO4-2, *Desulfonatronum thioautotrophicum* ASO4-1, *Desulfonatronum thiodismutans* MLF1, *Desulfonatronum lacustre* DSM 10312, *Desulfovibrio ferrophilus* IS5, *Desulfovibrio subterraneus* ND17, *Desulfovibrio mangrove* FT415, *Desulfovibrio desulfuricans* L4, *Desulfovibrio fairfieldensis* CCUG 45958, *Candidatus Desulfovibrio trichonymphae* Rs-N31. 120 bacterial marker genes were extracted and aligned from genome assemblies using GTDB-Tk 2.3.2 (42) with the GTDB Release 214. The maximum-likelihood phylogenetic tree was generated using IQ-TREE 2.3.4 (41) with LG+I+R4 model. Bootstraps are depicted at nodes, estimated by IQ-TREE (-B 1000 option). The scale bar indicates the number of substitutions per site.

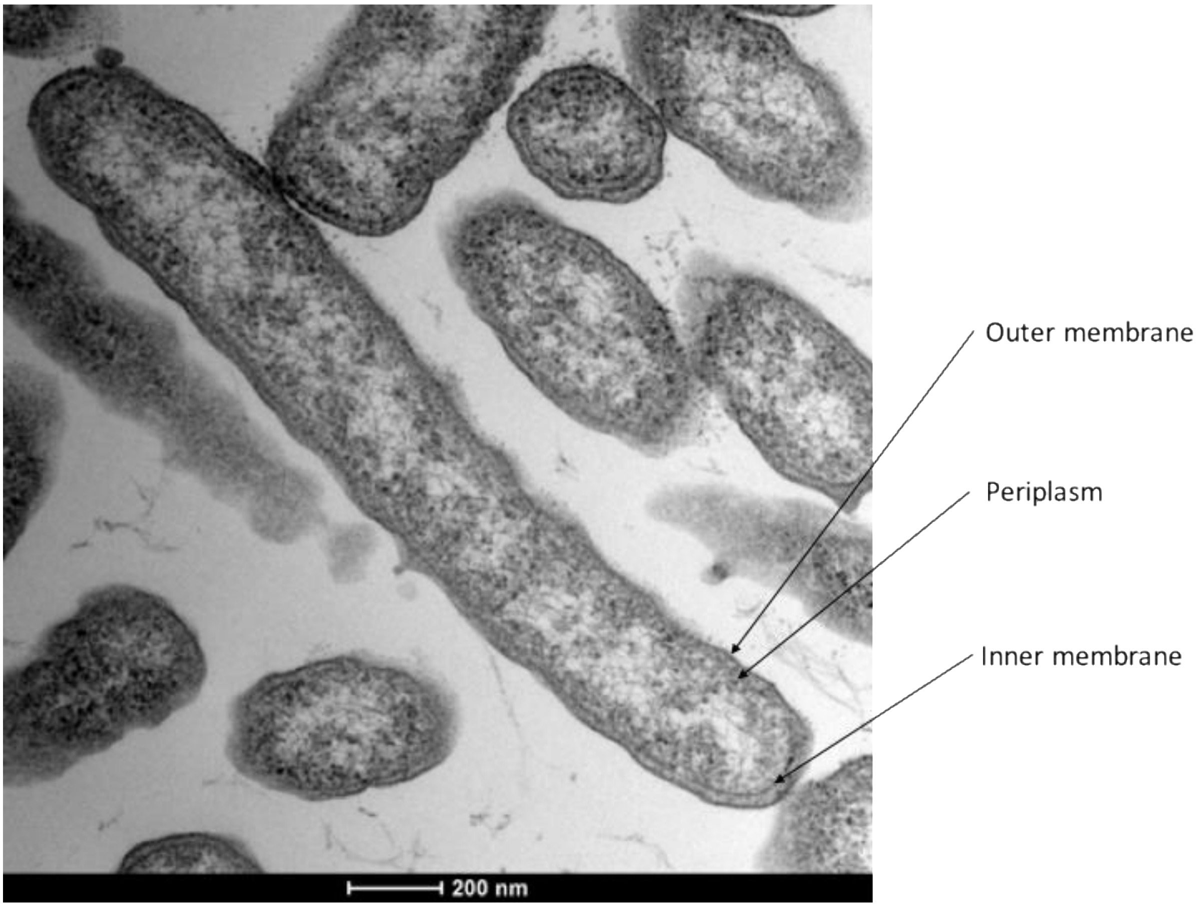

Figure 3. Transmission Electron Microscopy of strain V6Fe1^T grown on fumarate 20 mM and yeast extract 2 g/L showing a Gram-negative structure. Average width 0.2-0.4 µm, average length 4-8 µm.

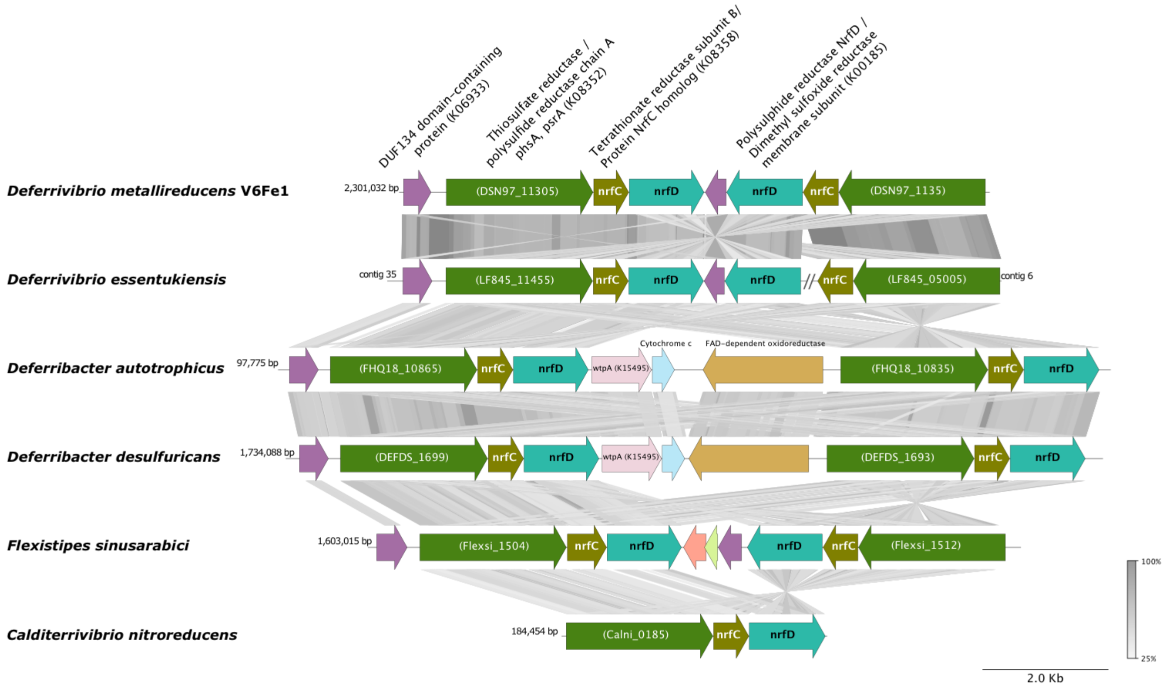

Figure 4. Comparative genome analysis of NrfD complex region among *Deferrivibrio metallireducens* V6Fe1^T and its closest relatives. CDS are represented by arrows indicating their orientation (direct or complementary strand) in the genome. CDS numbers are shown below the corresponding arrow according to the annotation by the CDS, which at MaGe (https://mage.genoscope.cns.fr/). The figure was generated using pyGenomeViz v1.0.0. The degree of amino acid similarity between paired ORFs was analyzed by tBLASTx search, and the percentage of similarity is represented in color from pale gray to dark gray. Note that the duplication plus inversion concerns Strain V6Fe1^T, *D. essentukiensis,* and *F. sinusarabici* genomes; *D. desulfuricans* and *D. autitrophicus* present a duplication without inversion, *C. nitroreducens* present no duplication.

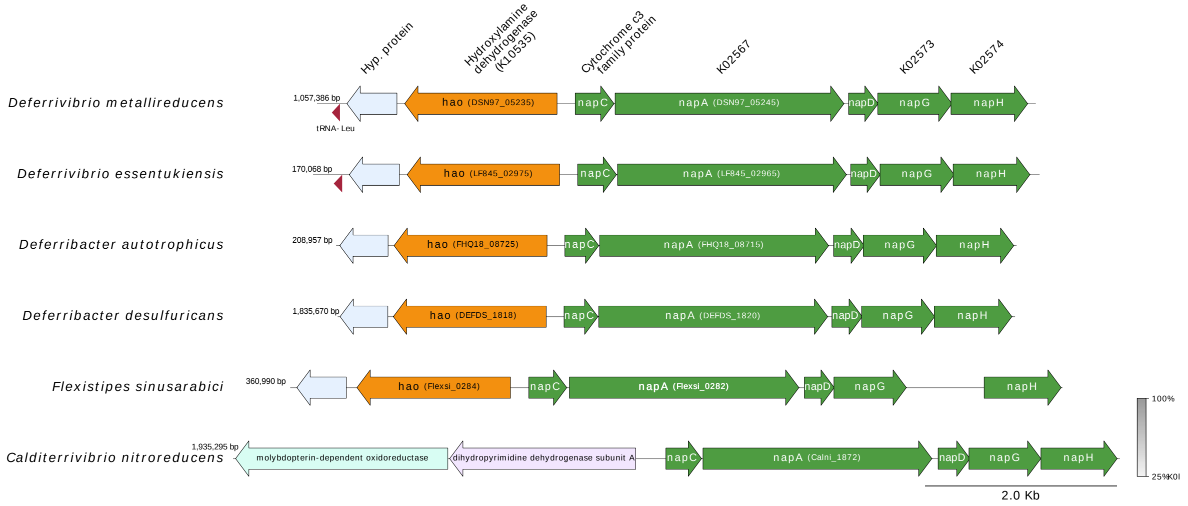

Figure 5. Comparative genome analysis of the region encoding the NapA complex genes of *Deferrivibrio metallireducens* V6Fe1^{T} and its closest relatives. CDS are represented by arrows indicating their orientation (direct or complementary strand) in the genome. CDS numbers are shown below the corresponding arrow according to the annotation by MaGe (https://mage.genoscope.cns.fr/). The figure was generated using pyGenomeViz v1.0.0. The degree of amino acid similarity between paired ORFs was analyzed by tBLASTx search, and the percentage of similarity is represented in color from pale gray to dark gray.

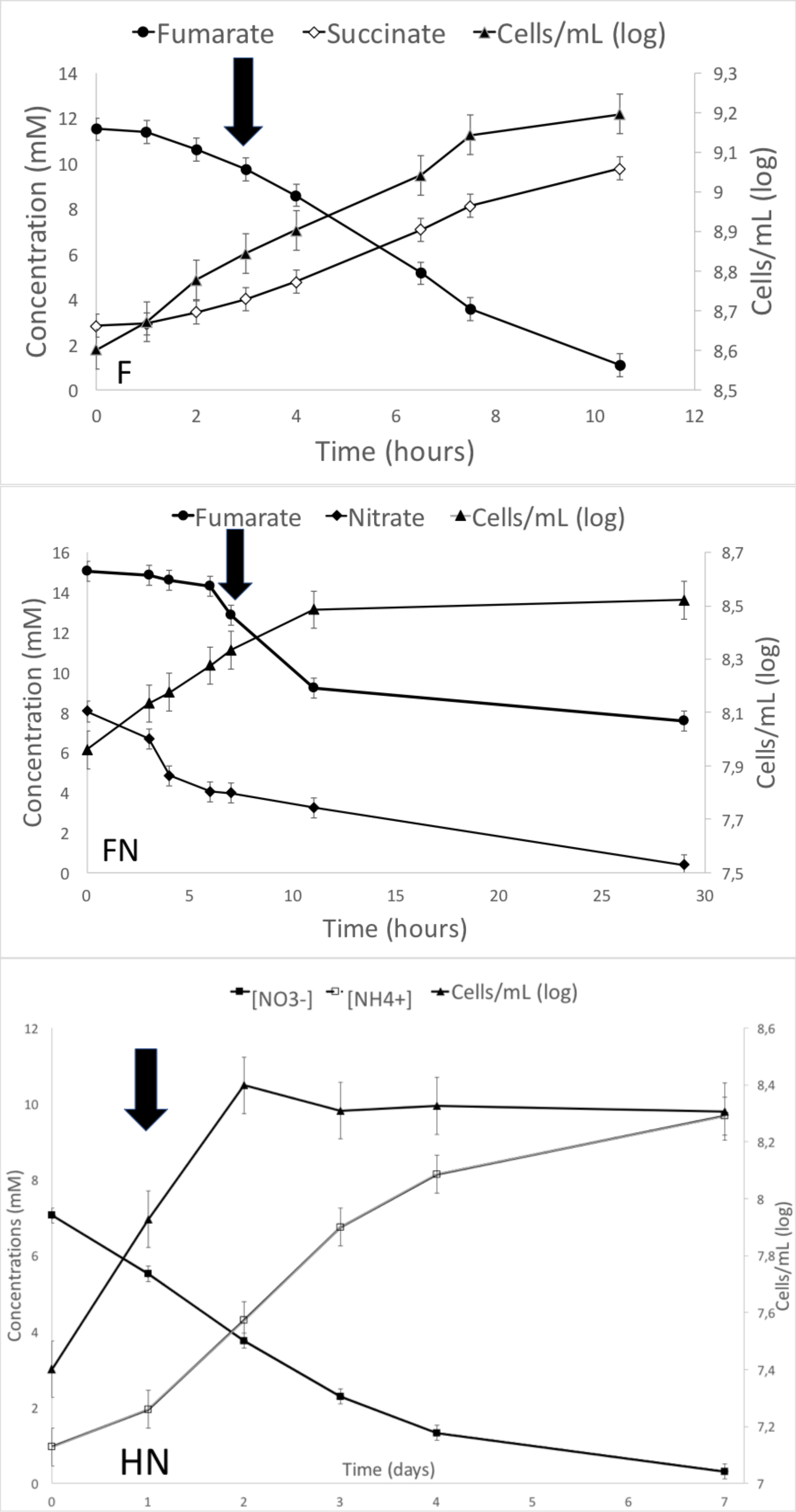
Fumarate
Succinate
Cells/mL (log)
Concentration (mM)
Cells/mL (log)
Time (hours)
F
Fumarate
Nitrate
Cells/mL (log)
Concentration (mM)
Cells/mL (log)
Time (hours)
FN
[NO3-]
[NH4+]
Cells/mL (log)
Concentrations (mM)
Cells/mL (log)
Time (days)
HN

Figure 6. Growth of strain V6Fe1^T by fermentation or nitrate reduction. Three conditions were tested: a) F: fermentation on Fumarate alone used as the reference condition; b) FN: respiratory nitrate reduction with fumarate as substrate; c) HN: nitrate reduction in autotrophic conditions with hydrogen as the sole electron donor. Cultures were performed in triplicates, and error bars represent the standard deviation. The black arrow represents the time of sampling for RT qPCR analyses.

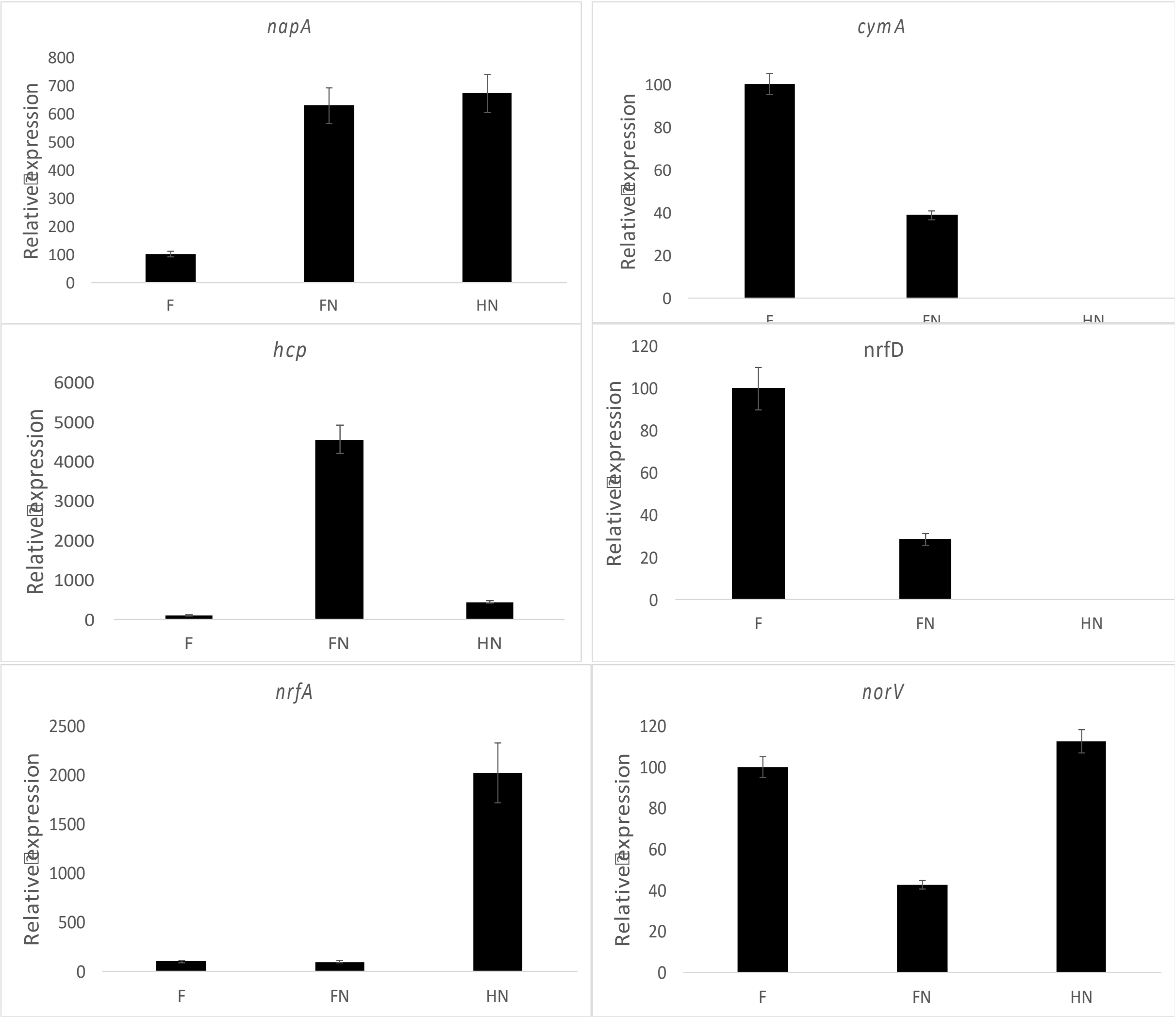

Figure 7. Expression profiles of genes involved in nitrate reduction depending on the substrate. The quantitative RT-PCR analyses were conducted on strain V6Fe1T at the exponential growth phase under different culture conditions. F represents the Fumarate control condition (100% expression), FN represents culture conditions with fumarate and nitrate, and HN represents the autotrophic culture condition with hydrogen as the sole electron donor and Nitrate. The barplots represent differential expression of *nap*A (periplasmic nitrate reductase), *cym*A (tetraheme c-type cytochrome involved in anaerobic respiration), *hcp* (hybrid cluster protein, possibly involved in hydroxylamine reduction or nitric oxide detoxification), *nrf*D (transmembrane protein involved in energy conservation), *nrf*A (formate-dependent nitrite reductase), and *nor*V (automatically annotated as anaerobic nitric oxide reductase flavorubredoxin).

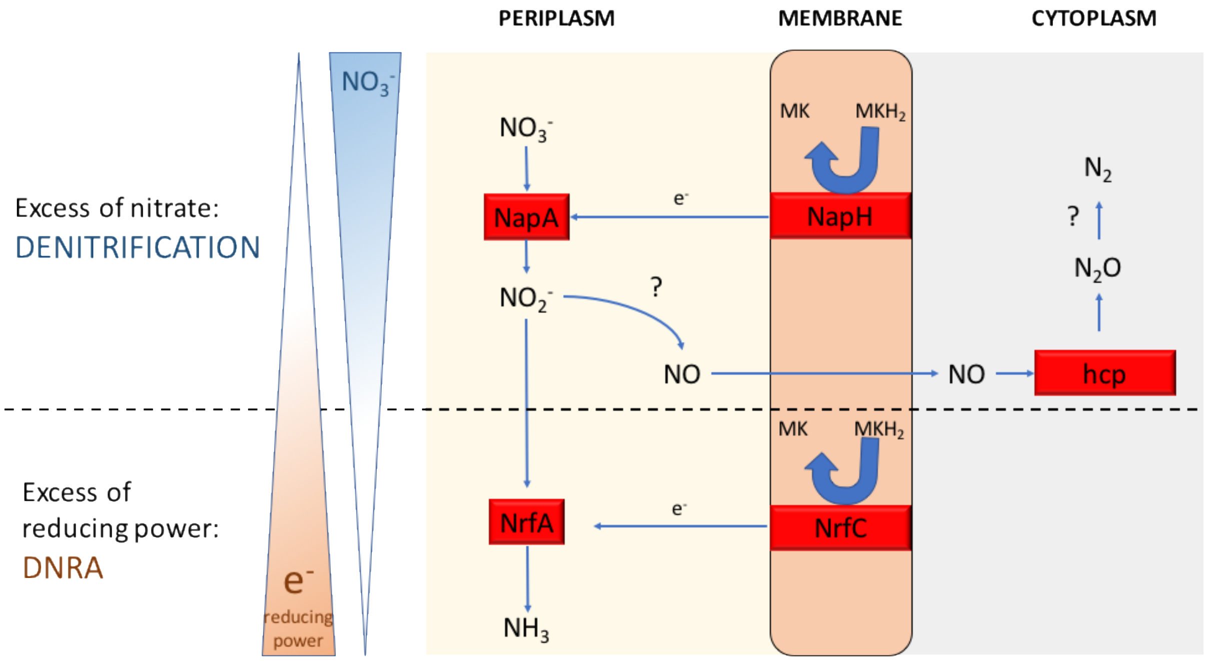

Figure 8. The hypothetical enzymatic nitrate reduction system in strain V6Fe1^T according to the nitrate/electron donor balance. Strain V6Fe1^T is unique within the *Deferribacterota* phylum by its ability to perform denitrification. NapA is the soluble periplasmic nitrate reductase characterized by its strong affinity for nitrate. We hypothesize that in case of excess of reducing power, strain V6Fe1^T performs DNRA via a NrfA protein; and that in case of shortage of reducing power, or nitrate excess, strain V6Fe1^T performs denitrification. Proteins involved in denitrification process still deserve investigation.

Click here to access/download
Supporting Information
Supplementary_Fig_Tab_juillet2024_PlosOne.docx
