# Supplementary material for: Metabolic versatility and nitrate reduction pathways of a new thermophilic bacterium of the Deferrivibrionaceae: Deferrivibrio metallireducens sp. nov isolated from hot sediments of Vulcano Island, Italy
Source: PLoS One. 2025 Mar 11;20(3):e0315093. doi: 10.1371/journal.pone.0315093 (PMC11896075; doi:10.1371/journal.pone.0315093)
Supplement: Attachment — Submitted filename: Rebuttal_Letter_PlosOne.docx [file pone.0315093.s003.docx]

Grégoire Galès	Assistant Professor	Marseille, October 28th, 2024

Gaël Erauso	Professor

Aix Marseille Université, CNRS,

Université de Toulon, IRD, OSU Pythéas,

Mediterranean Institute of Oceanography (MIO),

UM 110, 13288 Marseille, France

Dear Professor Appanna,

I am grateful to you and the two reviewers for your contributions. As a result, we have improved the quality and relevance of our article. The publisher and the two reviewers were correct in noting that the results and discussion section was too long and speculative. We have reduced the article by two or three pages and withdrawn ten references. We have focused on what we can demonstrate and eliminated hand-waving. We are confident you will find the corrections to the manuscript convincing.

You will find the answers to your questions and comments here, as well as in the text and figures. We have made the document easier to read by using bold and underlined text for our answers.

1. Please ensure that your manuscript meets PLOS ONE's style requirements, including those for file naming. The PLOS ONE style templates can be found at

https://journals.plos.org/plosone/s/file?id=wjVg/PLOSOne_formatting_sample_main_body.pdf and

<https://journals.plos.org/plosone/s/file?id=ba62/PLOSOne_formatting_sample_title_authors_affiliations.pdf>

**We have taken great care to ensure that the manuscript will meet your journal's expectations.**

2. Please include a complete copy of PLOS’ questionnaire on inclusivity in global research in your revised manuscript. Our policy for research in this area aims to improve transparency in the reporting of research performed outside of researchers’ own country or community. The policy applies to researchers who have travelled to a different country to conduct research, research with Indigenous populations or their lands, and research on cultural artefacts. The questionnaire can also be requested at the journal’s discretion for any other submissions, even if these conditions are not met. Please find more information on the policy and a link to download a blank copy of the questionnaire here: https://journals.plos.org/plosone/s/best-practices-in-research-reporting. Please upload a completed version of your questionnaire as Supporting Information when you resubmit your manuscript.

**This section is irrelevant to our manuscript. We simply benefited from the help of Italian colleagues, who are mentioned in the acknowledgments.**

3. In your Methods section, please provide additional information regarding the permits you obtained for the work. Please ensure you have included the full name of the authority that approved the field site access and, if no permits were required, a brief statement explaining why.

**No permits were required, as Italy is not a signatory to the Nagoya Protocol. We introduced the M&M section by saying: “Although Italy is not a signatory to the Nagoya Protocol, we carried out the sampling with the authorization of our collaborators from the INGV Palermo (Italy), as stated in the acknowledgments.”**

**Indeed, we (Galès, Postec & Erauso) published last year a strain characterization in IJSEM from comparable samples:”** ***Marinitoga aeolica* sp. nov., a novel thermophilic anaerobic heterotroph isolated from a shallow hydrothermal field of Panarea Island in the Aeolian archipelago, Italy.”**

4. Please include a separate caption for each figure in your manuscript.

**It has been done.**

5. Please review your reference list to ensure that it is complete and correct. If you have cited papers that have been retracted, please include the rationale for doing so in the manuscript text, or remove these references and replace them with relevant current references. Any changes to the reference list should be mentioned in the rebuttal letter that accompanies your revised manuscript. If you need to cite a retracted article, indicate the article’s retracted status in the References list and also include a citation and full reference for the retraction notice.

**It has been done. No cited article has been retracted yet. As requested by the first reviewer, we have reduced the list of references (ten references withdrawn).**

Reviewer #1: The article by Grégoire et al. describes the isolation of a novel thermophilic bacterium.

The manuscript is of interest, and the data support the conclusions.

I only have minor recommendations:

- Figure 6, FN graph: It is difficult to discern between the groups. Please change one to an open box or something more recognizable.

**It has been done. Nitrate is shown in an open box. This makes the figure easier to understand.**

- Figure 7: The X axis is cut off for cymA

**It has been corrected.**

- Figure captions: What is your n value for the studies? Please list in figure captions (for all figures). For the TEM, was this a representative image? How many images were captured?

**All experiments were performed in triplicate. About 25 TEM images were taken, from which we selected Figure 3. For the RT-qPCR experiments, three dilutions were analyzed. These clarifications have been included in the text and figure legends.**

- The Results and Discussion section is quite long (90 references) and contains a lot of hand-waving. For example, you discuss the roTCA cycle, and other enzymes that “could be” at work, but provide no functional evidence for many of them. I recommend trimming this section down by 2-3 pages and focusing on what was discovered and not what you believe may be happening

**The Results and Discussion section has been significantly reduced, as the reviewer asked. We have removed all sections deemed too speculative. The relevant section is now more than two pages shorter than initially planned, which improves the manuscript. Ten references have been withdrawn. We have eliminated the lengthy discussion of the roTCA cycle and the denitrification/DNRA switch regulation, which will be the subject of further research.**

Reviewer #2: Dear Editor,

I have thoroughly reviewed the manuscript entitled "Metabolic versatility and nitrate reduction pathways of a new thermophilic bacterium of the Deferrivibrionaceae: Deferrivibrio metallireducens sp. nov. isolated from hot sediments of Vulcano Island, Italy," and I would like to offer the following comments for improvement: I have also mentined the some changes in the MS pdf.

*Please remove any unnecessary paragraphs from the abstract for better clarity.

**There have been none in accordance with the wishes of the first reviewer.**

*Ensure consistent formatting for temperature symbols (Celsius) throughout the manuscript.

**It has been done throughout the text.**

*Properly align the tables and address any missing entries.

**This has been done.**

*Avoid the use of capitalized words inappropriately throughout the manuscript. Additionally, the manuscript requires English language corrections.

**This has been corrected.**

*Ensure proper referencing style and maintain consistency in the reference format.

**This has been done.**

*Italicize all scientific names and follow the standard rules of scientific nomenclature.

**This has been done.**

*In the Materials & Methods section, include the concentrations of the PCR components and provide details of the PCR programs used.

**This has been done.**

**In the text: “Quantitative PCR was performed with 2 μL cDNA (directly reverse transcribed DNA and 10-1/10-2 dilutions), 0.25 μL of each 10 μM primer, 10 μL of 2X SyberGreen and 7.5 μL of ultrapure RNAse/DNAse-Free distilled water (Thermofisher). Thermal cycling conditions were as follows: 2 min at 98°C, followed by 30 cycles of 5 s at 98°C, 30 s at 61°C, and 20 s at 72°C. Data collection was performed during each annealing phase.”**

*Consider using iTOL tools for better representation of the phylogenetic analysis figure.

**Thank you for your valuable feedback regarding the phylogenetic analysis figures. We want to clarify that the phylogenetic trees presented in Figures 1 and 2 have already been generated using iTOL. To enhance clarity and provide a more detailed response to the reviewer, we have updated the legends for both figures accordingly.**

*Correct any grammatical and language errors throughout the manuscript.

**This has been done, we hope our efforts will be seen and appreciated.**

We hope you will enjoy reading the corrections to this article. If you have any questions, please don’t hesitate to contact us.

The corresponding authors

Grégoire Galès

[gregoire.gales@univ-amu.fr](mailto:gregoire.gales@univ-amu.fr)

Gaël Erauso

[gael.erauso@univ-amu.fr](mailto:Gael.erauso@univ-amu.fr)
